# Bioengineering CXCR4-overexpressing cell membrane functionalized ROS-responsive nanotherapeutics for targeting cerebral ischemia-reperfusion injury

**DOI:** 10.7150/thno.60785

**Published:** 2021-07-06

**Authors:** Li Luo, Guangchao Zang, Boyan Liu, Xian Qin, Yuchan Zhang, Yidan Chen, Haijun Zhang, Wei Wu, Guixue Wang

**Affiliations:** 1 Key Laboratory for Biorheological Science and Technology of Ministry of Education, State and Local Joint Engineering Laboratory for Vascular Implants, Bioengineering College of Chongqing University, Chongqing 400030, China; 2 Laboratory of Tissue and Cell Biology, Lab Teaching & Management Center, Chongqing Medical University, Chongqing, 400016, China; 3 Institute of Life Science, Chongqing Medical University, Chongqing, 400016, China; 4 Department of Vascular & Intervention, Tenth Peoples' Hospital of Tongji University, Shanghai, 200072, China

**Keywords:** smart biomimetic nanoparticles, CXCR4/SDF-1 specific, ROS-responsive, ischemia-reperfusion injury, free radical scavenging

## Abstract

**Rationale:** As a potentially life-threatening disorder, cerebral ischemia-reperfusion (I/R) injury is associated with significantly high mortality, especially the irreversible brain tissue damage associated with increased reactive oxygen radical production and excessive inflammation. Currently, the insufficiency of targeted drug delivery and “on-demand” drug release remain the greatest challenges for cerebral I/R injury therapy. Bioengineered cell membrane-based nanotherapeutics mimic and enhance natural membrane functions and represent a potentially promising approach, relying on selective interactions between receptors and chemokines and increase nanomedicine delivery efficiency into the target tissues.

**Methods:** We employed a systematic method to synthesize biomimetic smart nanoparticles. The CXCR4-overexpressing primary mouse thoracic aorta endothelial cell (PMTAEC) membranes and RAPA@HOP were extruded through a 200 nm polycarbonate porous membrane using a mini-extruder to harvest the RAPA@BMHOP. The bioengineered CXCR4-overexpressing cell membrane-functionalized ROS-responsive nanotherapeutics, loaded with rapamycin (RAPA), were fabricated to enhance the targeted delivery to lesions with pathological overexpression of SDF-1.

**Results:** RAPA@BMHOP exhibited a three-fold higher rate of target delivery efficacy via the CXCR4/SDF-1 axis than its non-targeting counterpart in an *in vivo* model. Additionally, in response to the excessive pathological ROS, nanotherapeutics could be degraded to promote “on-demand” cargo release and balance the ROS level by *p*-hydroxy-benzyl alcohol degradation, thereby scavenging excessive ROS and suppressing the free radical-induced focal damage and local inflammation. Also, the stealth effect of cell membrane coating functionalization on the surface resulted in extended circulation time and high stability of nanoparticles.

**Conclusion:** The biomimetic smart nanotherapeutics with active targeting, developed in this study, significantly improved the therapeutic efficacy and biosafety profiles. Thus, these nanoparticles could be a candidate for efficient therapy of cerebral I/R injury.

## Introduction

Ischemic stroke is one of the leading causes of morbidity and mortality around the world 1. After a stroke, restoration of perfusion is the most frequent and effective medical treatment, but the process may inflict ischemia-reperfusion (I/R) injury 2. Oxidative stress is a prevalent feature of stroke I/R, resulting in calcium overload, glial cell swelling, endothelial dysfunction, free radical production, and inflammation 3-5. These features may perpetuate a noxious excessive reactive oxygen species (ROS) microenvironment with irreversible damage to vital organelles and DNA 6-8, ultimately leading to cell apoptosis and necrosis 9,10.

The efficacy of current regimens with the single-agent therapy remains limited 11,12 and includes unfavorable biocompatibility, overly high dosages, and adverse effects 13,14. The introduction of cell membrane coating nanotechnology has brought a promising approach for cardiovascular and cerebrovascular disease treatment 15-19, such as the development of erythrocyte membrane- or neural stem cell (NSCs) membrane-coated nanoparticles (NPs) 20-22. Primary mouse thoracic aorta endothelial cells (PMTAEC) possess elastic and plastic biomechanical performance 23,24 with high purity and stem cell-like functional properties 25-29. Interestingly, the persistent overproduction of SDF-1 in I/R regions within the brain 30-32 resulted in CXCR4/SDF-1-driven chemokine signaling, leading to the accumulation and homing of endothelial cells at the site of injury 33-37. In this context, bioengineered CXCR4-overexpressing cell membrane-coated NPs exhibited disease-specific targeting with CXCR4/SDF-1-mediated accumulation 38. Furthermore, the low immunogenicity and long-circulating properties due to the CD47 protein on the membrane represented additional potential advantages 39,40.

Besides the enhanced targeted delivery, new approaches to enable free radical scavenging are urgently needed 41,42. Some reagents, such as CeO_2_, Prussian blue (PB), Pt, and NiO, exhibit a remarkable ROS-scavenging activity 43-45. Particularly, *p*-Hydroxybenzyl alcohol (HBA) has been used as an antioxidative stress and anti-inflammatory agent to achieve better therapeutic outcomes 46. As the exogenous ROS sources-responsive trigger 47, oxalyl chloride (OC) is usually employed for ROS-cleavable degradation 48. Therefore, ROS-based multicomponent structures can be integrated to construct the complex functionalization of NPs. In the ROS environment, the OC-conjugate can dissociate to accelerate cargo release, while the HBA degradation scavenges the excessive ROS.

The mTOR signaling pathway is known to play an active role in enhancing neuron survival by removing damaged cellular organelles during the I/R injury, especially drives pro-inflammatory M1 microglia toward an anti-inflammatory M2-like state and has emerged as a critical therapeutic agent for attenuating inflammation and alleviating cerebral I/R injury 49. Based on the above theory, rapamycin (RAPA), the mTOR inhibitor, loaded into the HOP nanocarriers (RAPA@HOP).

Herein, we chemically polymerized HBA with OC and poly-(ethylene glycol)_2000_ (PEG_2000_), generating ROS-responsive amphiphilic copolymer HBA-OC-PEG_2000_ (HOP). RAPA was loaded into HOP NPs, which were subsequently coated with the CXCR4=overexpressing bioengineered cell membranes formulating RAPA@BMHOP. This strategy offered several advantages: 1) RAPA@BMHOP displayed active and effective targeting, and the unique role of CXCR4/SDF-1-dependent signaling resulted in the high-efficiency accumulation of CXCR4 overexpression toward SDF-1 and involved SDF-1-mediated recognition; 2) As a major component for the recovery of normal physiological functions, RAPA@BMHOP exhibited ROS-responsive degradation by selectively controlling cargo release, playing a vital role in the dynamic balancing of cellular ROS, decreasing oxidative damage and neuroinflammation, and preventing subsequent damage to the injured area; 3) Besides its favorable pharmacokinetic profiles, the stealth effect and good biocompatibility could be attributed to cell membrane coating technology.

**Scheme 1:**
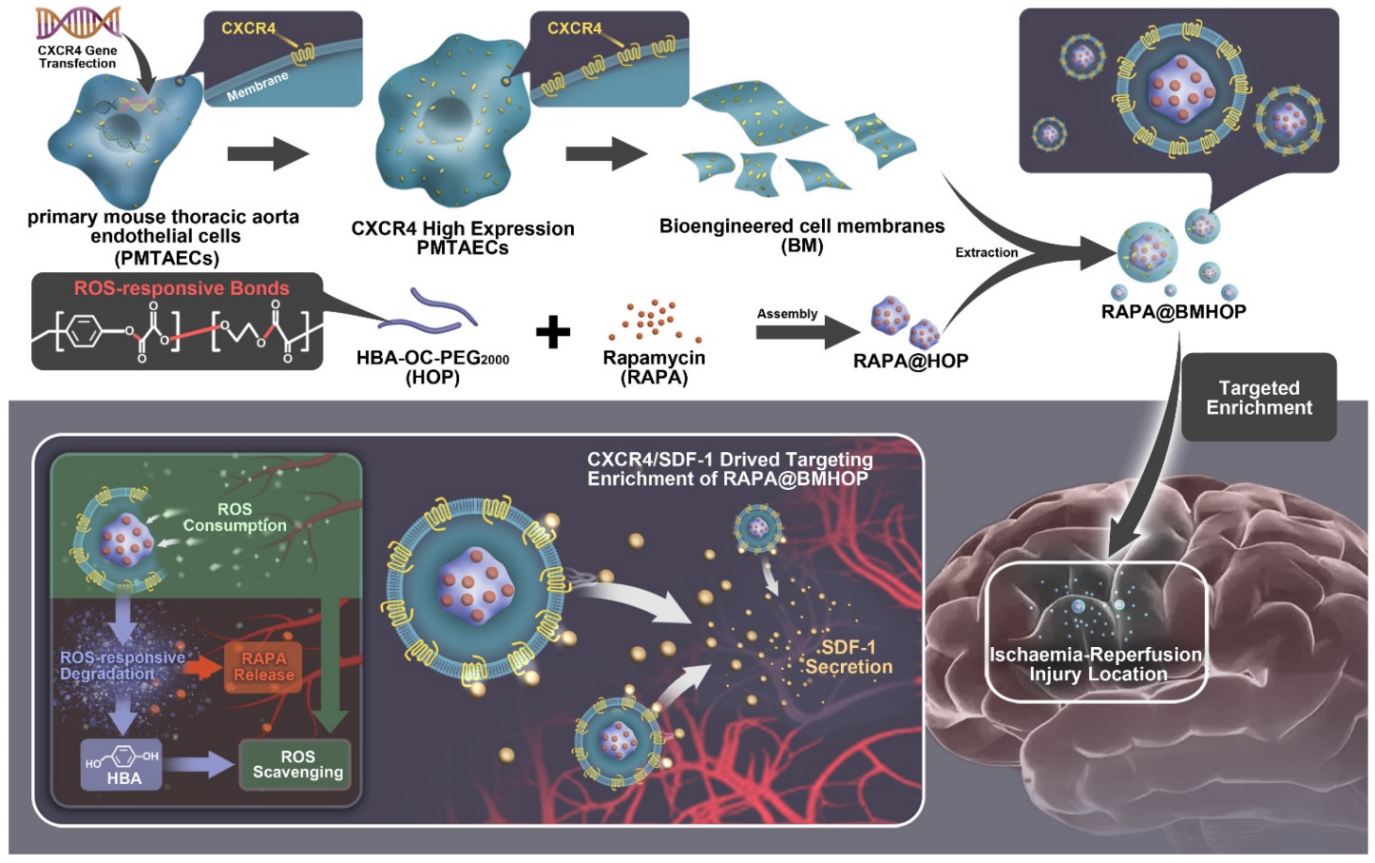
Illustration of bioengineered cell membrane-coated RAPA@BMHOP nanoparticle formation, including CXCR4-overexpressing cells obtained by lentiviral transfection and synthesis of HBA-OC-PEG_2000_. The smart nanomedicine RAPA@BMHOP was promoted to target cerebral I/R injury areas by interactions of CXCR4 with SDF-1, causing ROS-responsive cargo release within the oxidative microenvironment.

The endocytic internalization of RAPA@BMHOP by recipient cells is mediated by the CXCR4/SDF-1 mechanism, selectively delivering the nanocarriers to administer therapeutics to the excessive ROS intracellular sites. The oxalate bond of HBA-OC-PEG_2000_ cleavage leads to the RAPA release into the microenvironment. Thus, RAPA@BMHOP could be a feasible platform for achieving active delivery to ischemic areas and improving therapeutic efficacy for I/R injury (Scheme 1).

## Results and Discussion

### Characterization of bioengineered cell membrane-coated NPs

HOP was synthesized through a one-step condensation polymerization with OC. The harvested copolyoxalate was purified through repeated precipitation into cold diethyl ether and obtained as pale yellow transparent colloidal solid after drying under a high vacuum. The chemical structure of HOP was confirmed by ^1^H NMR (Figure S1).

**Figure 1:**
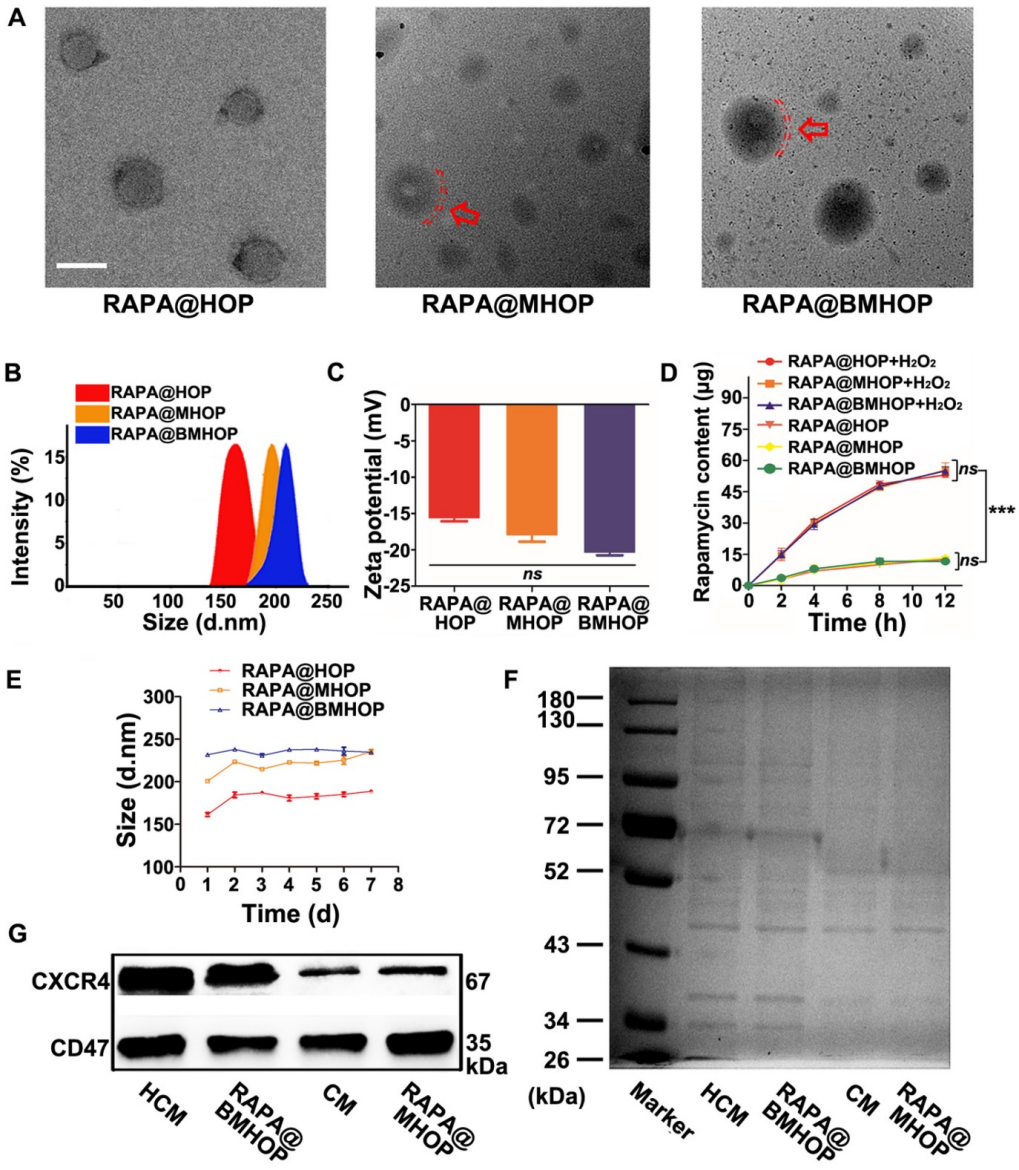
Characterization of bioengineered cell membrane-coated NPs. (A) TEM image of RAPA@HOP, RAPA@MHOP, and RAPA@BMHOP (scale bar = 200 nm; inside. red dashed circles represent the cell membrane). (B) Hydrodynamic size distribution and (C) ζ potential of RAPA@HOP, RAPA@MHOP, and RAPA@BMHOP (values represent mean ± SD, *n* = 3). (D) Drug release of different NPs and (E) Stability of NPs in 0.01 M PBS at room temperature (values represent mean ± SD, *n* = 3). (F) SDS-PAGE protein analysis and (G) Western blotting of indicated proteins.

RAPA loading (named RAPA@HOP) was achieved using a dialysis method. An extrusion method was used to prepare different NP formulations, including PMTAEC membrane-coated RAPA@HOP (RAPA@MHOP) and CXCR4-overexpressing PMTAEC membrane-coated RAPA@HOP (RAPA@BMHOP). The nanomaterial obtained by repeated passing through the extruder had several features, such as specific particle size distribution, relatively complete particle coating, high purity, and some degree of consistent lipid shells 17,50,51. Transmission electron microscopy (TEM) revealed spherical nanoparticles with the dehydrated size range of 170~200 nm for RAPA@HOP, 180~220 nm for RAPA@MHOP, and 180~230 nm for RAPA@BMHOP. Additionally, an outer layer was observed in RAPA@MHOP and RAPA@BMHOP samples (marked by the red arrows and red dashed circles), indicating the membrane-formed shell structure of the NPs (Figure 1A). Moreover, the average hydrodynamic diameter of RAPA@HOP was 172.5 nm; RAPA@MHOP and RAPA@BMHOP were slightly larger at 219.6 nm and 227.8 nm, respectively (Figure 1B). The ζ potentials of RAPA@MHOP and RAPA@BMHOP were measured as -18.1 mV and -20.5 mV, respectively, compared to RAPA@HOP with ζ potential of -15.7 mV (Figure 1C), as observed by dynamic light scattering (DLS). Changes in surface properties often lead to variations in nanoparticle size and surface electronegativity 52. The relatively larger particle sizes and surface ζ potentials of RAPA@MHOP and RAPA@BMHOP suggested that the membranes were successfully appended onto the surface of NPs 21,53,54.

During stroke I/R, over-production and accumulation of ROS triggered a rapid release of RAPA from the stimulated NPs. The physical stability of RAPA@HOP, RAPA@MHOP, and RAPA@BMHOP was compromised upon hydrolysis for 2 h in the presence of H_2_O_2_ (Figure S2, Figure S3A, and Figure 1D). Quantitative analysis revealed approximately 27.3% of RAPA at 2 h in 1 mM H_2_O_2_-containing phosphate-buffered saline (PBS). RAPA concentration gradually increased with time and reached the drug release limit point at 12 h (Figure S4). In the absence of H_2_O_2_, RAPA@HOP, RAPA@MHOP, and RAPA@BMHOP retained their stability without apparent size increase at 8 h. However, there was a slight release (13.2%) of the drug at the 8 h time point, although no significant RAPA accumulation was observed beyond 8 h, indicating their good stability and reduced premature cargo leakage during blood circulation.

To further evaluate the ROS-responsive behaviors of RAPA@HOP, RAPA@MHOP, and RAPA@BMHOP, their hydrolysis profiles were investigated in 0.01 M PBS (pH 7.4). All three NPs exhibited excellent stability without apparent changes in size for one week at room temperature, possibly attributed to the hydrophilic surface glycan layers in the phospholipid acid molecular layer cell membrane 48. For seven consecutive days, the average size of RAPA@HOP remained at 181.81 ± 9.16; RAPA@MHOP was 220.91 ± 11.16, and RAPA@BMHOP was approximately 236.4 ± 2.70. However, there were some significant changes in particle size at 37 °C after one week (Figure 1E), suggesting that nanocarrier formulations maintained better stability under relatively low temperatures. Particle size changes of RAPA@HOP, RAPA@MHOP, and RAPA@BMHOP in PBS with/without 1 mM H_2_O_2_ were monitored over time by DLS to test the physical stability of the cell membrane-coated NPs. Compared with the stable particle size of NPs in PBS, the sizes of NPs in H_2_O_2_-containing PBS gradually increased in 12 h, revealing their ROS-responsive degradation for an enhanced local cargo release (Figure S3B).

Bioengineered cells were collected for further experiments after lentiviral transduction of PMTAEC (Figure S5A-D and Figure S6). The proteins of the CXCR4-overexpressing PMTAEC membranes were analyzed by sodium dodecyl sulfate-polyacrylamide gel electrophoresis (SDS-PAGE). The cell membrane (CM) modification strategy rendered RAPA@BMHOP with similar exterior structures as CXCR4-overexpressing PMTAEC (Figure 1F). Extracted CM and CM-coated NP proteins showed highly consistent bands, matching closely with that of the whole PMTAEC, suggesting significant retention of CM proteins in RAPA@MHOP (Figure S7A-D). To further confirm the successful modification of specific targeting proteins on RAPA@BMHOP, representative adhesion protein CXCR4 was evaluated by Western blotting (WB). As shown in (Figure 1G), both CM and RAPA@BMHOP exhibited CXCR4 expression compared with lentivirus transfected PMTAEC (Figure S7B-D).

The ideal targeting NPs should be able to evade the human immune system. Endothelial cells widely express CD47 that can bind to the signal regulatory protein (SIRP) on the surface of macrophages, subsequently recruiting SHP-1 protein to produce a cascade of reactions to inhibit their phagocytosis by macrophages 55. WB analysis showed a CD47 band in all CM samples, RAPA@MHOP, CXCR4-overexpressing CM (HCM), and RAPA@BMHOP, demonstrating that the membrane coated onto the NPs, contained CXCR4 and CD47 and retained their functionality.

### Involvement of CXCR4/SDF-1 axis in targeted delivery and enhanced accumulation of nanotherapeutics in ischemic brain lesions

**Figure 2:**
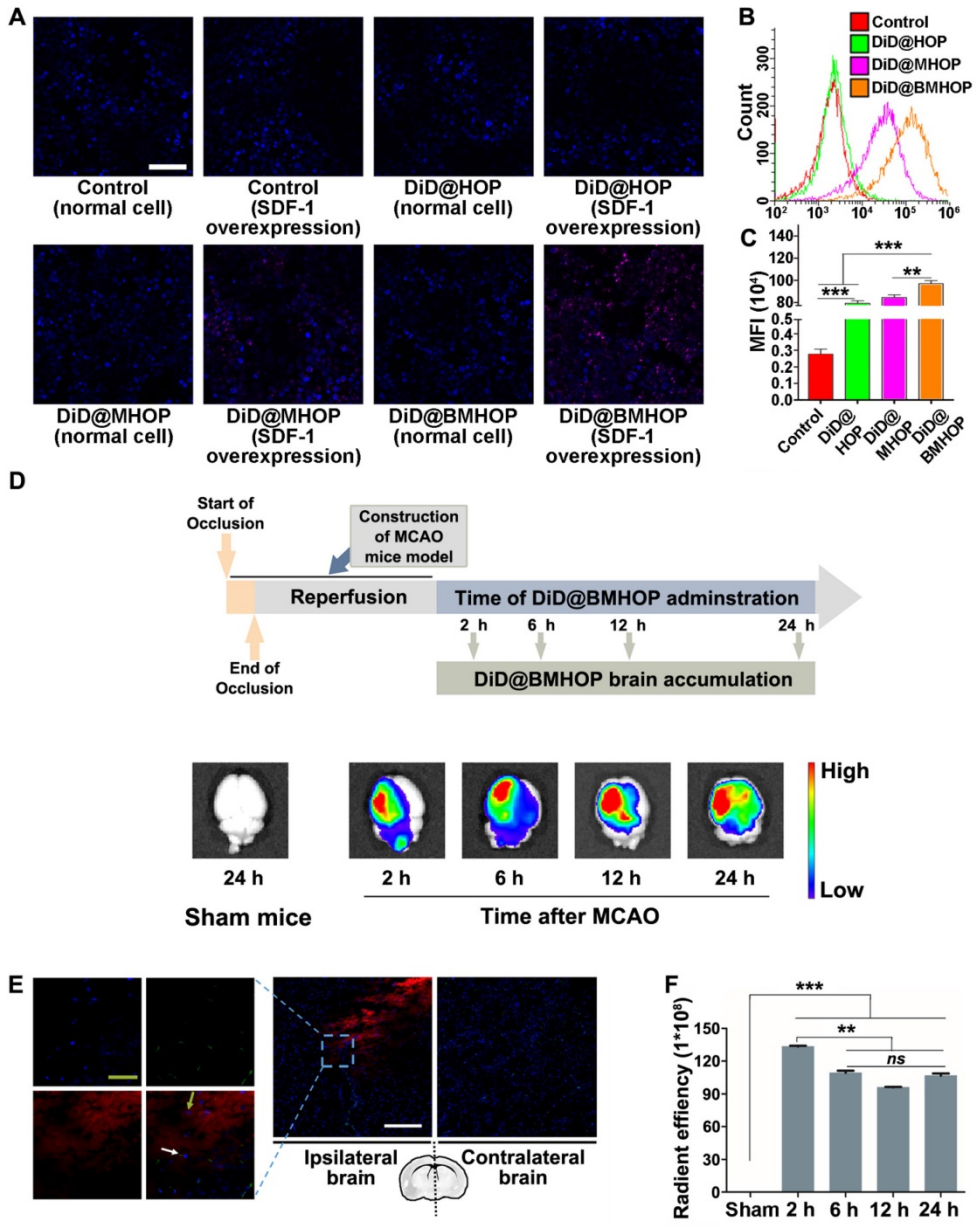
CXCR4 overexpression contributes to NP accumulation and homing in the ischemic microenvironment through interaction with SDF-1. (A) CLSM images of increased DiD@NPs accumulation in a cellular environment with SDF-1 overexpression (scale bar = 20 μm). (B) Flow cytometry results of the cellular uptake of DiD@NPs by SDF-1-overexpressing HUVECs. (C) Quantitative analysis of red fluorescence of different DiD@NPs (values represent mean ± SD, *n* = 3, **P < 0.01, ***P < 0.001). (D) *Ex vivo* IVIS imaging of targeted accumulation of DiD@BMHOP in the brain at different time points (2, 6, 12, and 24 h) after i.v. injection. Healthy mice in the control group were injected with DiD@BMHOP. (E) Distribution of DiD@BMHOP in ischemic penumbra (blue: DAPI for nucleus; green: CD31 for blood vessels; red: DiD@BMHOP). The white arrow indicates DiD@BMHOP associated with blood vessels; greyish-green arrow indicates DiD@BMHOP diffused away from blood vessels (white scale bar = 50 µm, greyish-green bar = 20 µm). (F) Quantification of the radiant efficiency of DiD@BMHOP in the brain by using IVIS Lumina imaging software performed by drawing a region of interest (ROI) (values represent mean ± SD, *n* = 3, **P < 0.01 and ***P < 0.001, *ns*, no significance).

Previous reports have shown that the homologous adhesion nature of cell membrane-cloaked platforms depended on surface antigens 18,56,57. Adding AMD3100 (potent and selective antagonist of the CXCR4 chemokine receptor) inhibited the accumulation of NPs to block the SDF-1-mediated interaction of CXCR4 58. A competitive inhibition study provided better evidence for targeting NP accumulation *in vitro* (Figure S8A-C). Theoretically, the primary mode of action of RAPA@BMHOP would be via specific CXCR4/SDF-1 targeting to an SDF-1-overexpressing microenvironment 59. To evaluate the ability of NPs to specifically target SDF-1-overexpressing cells, human umbilical venous endothelial cells (HUVECs) were treated with NPs (RAPA-loaded NPs were substituted with DiD encapsulation for fluorescence analysis) before or after oxLDL-stimulation (overexpression of SDF-1) 60. The results showed that DiD@BMHOP preferentially accumulated in oxLDL-stimulated HUVECs (Figure 2A). Similarly, oxLDL-induced HUVECs exhibited significantly stronger DiD@BMHOP fluorescence than control cells, DiD@HOP, and DiD@MHOP, as measured by quantitative flow cytometry (Figure 2B). In contrast with normal NPs, a 72.4-fold increase in DiD@BMHOP accumulation was observed when HUVECs were incubated with NPs for 2 h (Figure 2C), indicating the involvement of a CXCR4/SDF-1 axis in enhanced assimilation of RAPA@BMHOP *in vitro* 61.

We evaluated whether CXCR4-overexpressing cell membrane improved targeted delivery of NPs by studying NPs accumulation in the ischemic brain shortly after i.v. administration of NPs encapsulating an infrared fluorescent DiD dye. Among three different NPs, DiD@BMHOP showed significant accumulation in the ischemic brain area compared with the control DiD@HOP group with no cell membrane coating or DiD@MHOP NPs with low CXCR4-targeting ability (Figure S9). Compared with images of the contralateral left brain and healthy mice, IVIS Lumina II imaging (Figure 2D) confirmed targeted accumulation of DiD@BMHOP in the ischemic side within the first 2 h after i.v. administration of NPs. Fluorescence imaging showed the presence of red DiD at 6 h after the i.v. injection with strong fluorescence in lesion sites at 12 h and 24 h post-injection with DiD@BMHOP. The targeted delivery of NPs is expected to maximize the treatment efficacy of cerebral I/R injury while minimizing undesirable side effects 62-64. We further investigated the distribution of DiD@BMHOP within the ischemic area. Co-localization of CD31 immunostaining in brain sections and red fluorescence images indicated the availability of the nanodrug DiD@BMHOP in the re-perfused injured brain location (Figure 2E and Figure S10) that could diffuse into the brain parenchyma 65. Because of the SDF-1 infiltration in the ischemic brain area, DiD@BMHOP could diffuse from blood vessels and accumulate in the lesion driven by the chemokine-ligand (CXCR4/SDF-1) interaction.

Consistent with a previous report 66, the red fluorescence signal in the ipsilateral brain hemisphere showed enhanced DiD@BMHOP accumulation compared with the contralateral side with a low expression level of SDF-1. Quantification of the fluorescence intensity in the brain indicated that DiD@BMHOP accumulation significantly increased when injected at the early stage I/R injury with a decreased fluorescence intensity when the nanodrug was injected at 6, 12, or 24 h post-surgery (Figure 2F). Even so, the fluorescence signal, indicating a wider biodistribution of DiD@BMHOP, was still present in the brain by 24 h post-injection. These results indicated prolonged brain accumulation of NPs for 24 h following administration via the tail vein, suggesting their promising potential for sustained drug release.

### Nanodrug with ROS-sensitivity for excess ROS scavenging

**Figure 3:**
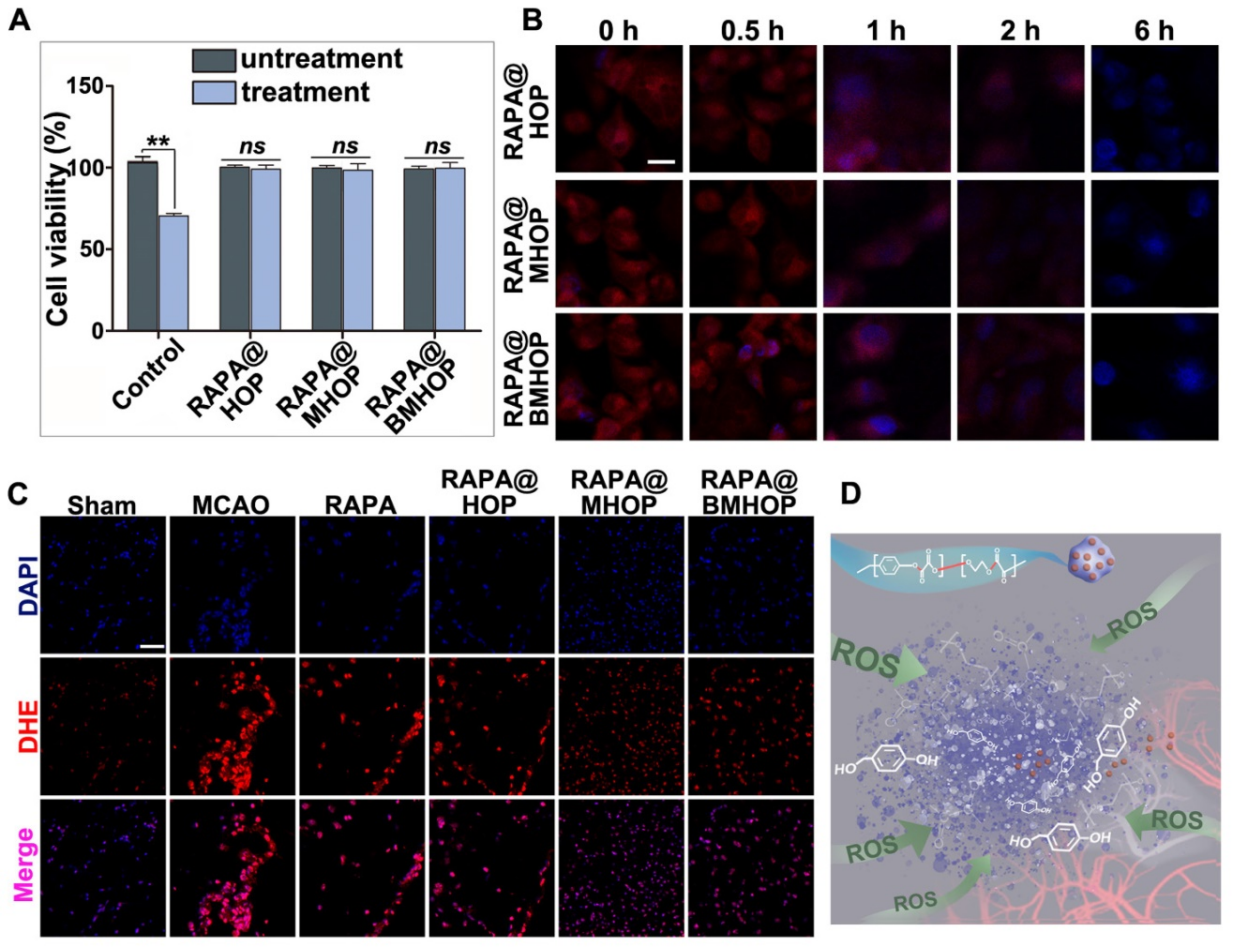
Overproduced ROS elimination accompanied by ROS-induced polymeric degradation. (A) Effects of RAPA@BMHOP on H_2_O_2_-induced HUVECs and untreated HUVECs (values represent mean ± SD, *n* = 6, **P < 0.01, *ns*, no significance). (B) CLSM images from three different RAPA@NPs on intracellular ROS elimination at different time points (scale bar = 5 μm). (C) CLSM images of red fluorescent signals of DHE for detecting ROS levels in the ischemic brain (scale bar = 20 μm). (D) Schematics of controlled drug release from ROS facilitating breakage of active chemical bonds.

Treatment of HUVECs with H_2_O_2_ (300 μM) has been shown to significantly increase ROS production 67. Notably, RAPA@BMHOP (5 μg/mL) suppressed H_2_O_2_-induced cytotoxicity, and RAPA@HOP or RAPA@MHOP (5 μg/mL) also produced a similar effect. As shown in (Figure 3A), compared with untreated HUVECs, treatment of cells with RAPA@NPs significantly restored cell viability, suggesting that RAPA@NPs attenuated intracellular ROS level and suppressed H_2_O_2_-induced oxidative stress. Furthermore, all nano-formulations could significantly restore cell viability by the active HBA resulting from HBA-OC-PEG_2000_ degradation, ultimately reversing H_2_O_2_-induced cytotoxicity in cells. Therefore, the inhibitory effects of RAPA@NPs on ROS generation merits further investigation. To examine the ROS scavenging ratio of RAPA@NPs, intracellular ROS levels were detected with 10 μM dihydroethidium (DHE). CLSM results illustrated that RAPA@BMHOP (5 μg/mL) significantly inhibited ROS generation in a time-dependent manner. As displayed in (Figure 3B), intracellular ROS levels and the red fluorescence in the cells were decreased 1 h after treatment. The intensity of red fluorescence reduced and almost could not be visualized after 6 h of treatment with RAPA@BMHOP. Further, fluorescence images of DHE-stained cells indicated consumption of intracellular ROS accompanied by decreased red fluorescence in the cells, consistent with flow cytometry results (Figure S11A-B).

By comparison, the cells pre-treated with H_2_O_2_ experienced a significant drop in ROS concentration upon treatment with HBA-incorporated polyoxalate NPs, indicating the scavenging of ROS by NPs *in vitro*. Given the strong antioxidative property of RAPA@BMHOP, we examined their potential capability in scavenging ROS *in vivo*. As shown in (Figure 3C), compared to MCAO and other groups, the DHE fluorescent probe showed the highly efficient ROS-eliminating potential of RAPA@BMHOP *in vivo* manifested by markedly reduced ROS levels in frozen sections of the brain. A major challenge in I/R injury is the ischemic brain injury risk due to high ROS levels that induce cell dysfunction and cell death within the neurovascular unit 68. Elimination of increased intracellular ROS levels and inhibition of ROS generation are critical therapeutic approaches for preventing free radical damage and repairing DNA strand breakage in the ischemic brain region 69. Overwhelming ROS production could play a key role in the breakage of peroxalate ester linkages (Figure 3D). In an *in vivo* pathological situation, a dynamic state of equilibrium of cellular ROS could occur in the brain by pharmacological therapies, enabling the recovery of damaged cells. When RAPA@BMHOP was delivered to specific sites, the ROS-cleavable oxalate bond of HBA-OC-PEG_2000_ led to drug release into the microenvironment through the consumption of excessive ROS. Thus, the lesion site showed a tendency for recovery to normal ROS level following treatment with RAPA@BMHOP.

### Brain protection of RAPA@BMHOP from MCAO-induced I/R injury

**Figure 4:**
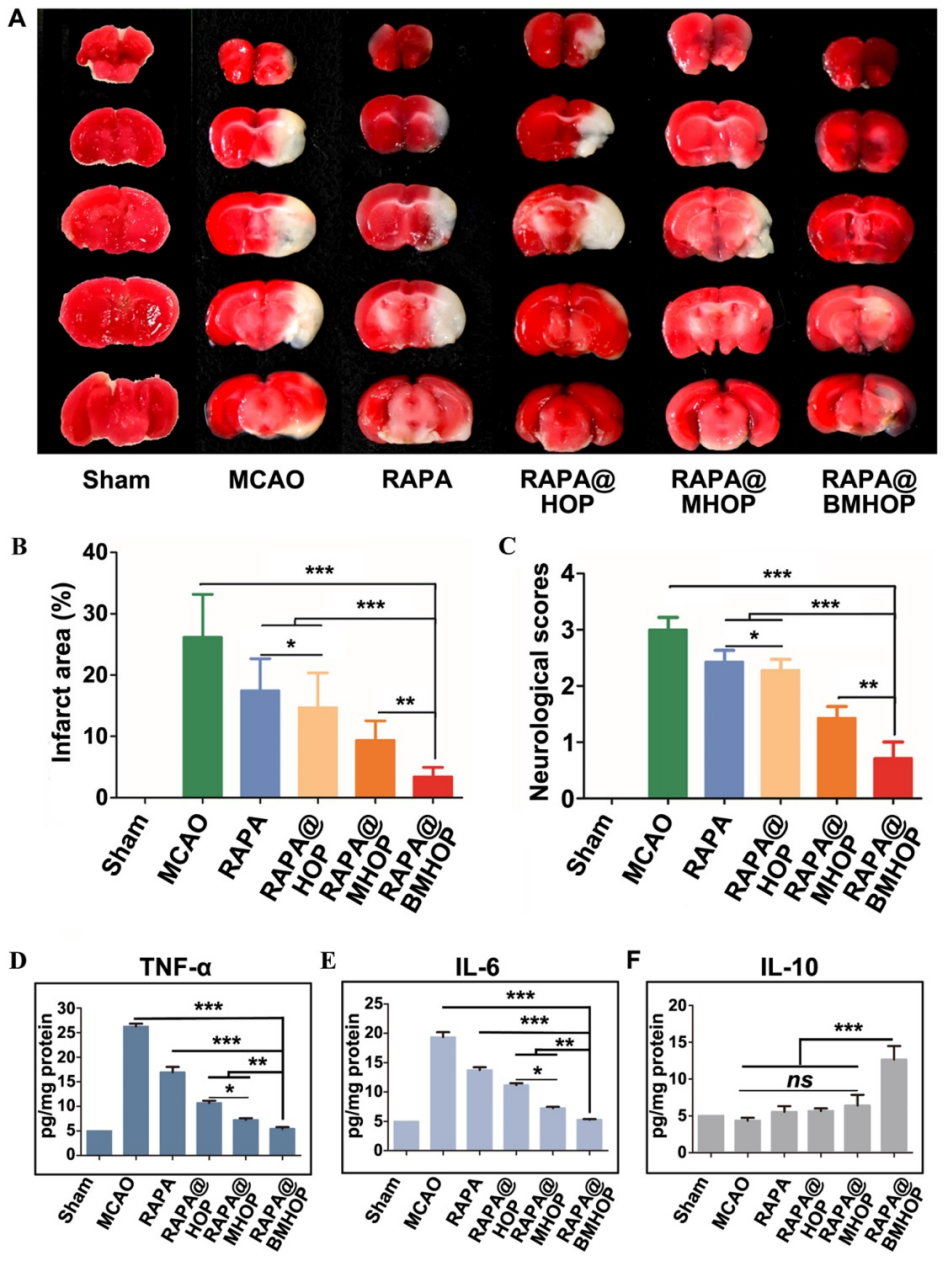
Treatment efficacy of RAPA@NPs for cerebral I/R injury. (A) Representative TTC-stained brain sections of Sham-operated and MCAO (group administered with 5% sucrose), RAPA@HOP, RAPA@MHOP, RAPA@BMHOP groups. The nonischemic region is shown as red, and the infarction region is shown as white. (B). Quantification of brain infarct volume in different mice groups and (C) mean neurological scores of MCAO mice after the treatments (values represent mean ± SD, *n* = 3, *P < 0.05, **P < 0.01 and ***P < 0.001). A higher score indicates a more severe injury. Statistical analysis performed by two-way ANOVA test. (D-F) Relative expression of pro-inflammatory (TNF-α and IL-6) and anti-inflammatory factors (IL-10) in brain tissues (values represent mean ± SD, *n* = 3, *P < 0.05, **P < 0.01 and ***P < 0.001, *ns*, no significance).

When the MCAO mice brain infarction area was stained by using the triphenyl tetrazolium chloride (TTC) assay, compared to the large ischemic area in the MCAO group, RAPA@BMHOP was found to exert a protective effect on the salvageable ischemic penumbra (Figure 4A). An evaluation of the protective effects with 0.05 mg/kg of RAPA@HOP, RAPA@MHOP, and RAPA@BMHOP showed the most significant decrease of the infarction area (10.7 ± 1.3%) in MCAO mice treated with RAPA@BMHOP to, compared to other NPs, suggesting better recovery of ischemic injuries with RAPA@BMHOP (Figure 4B). The neuroscores were also evaluated at 72 h following treatment. RAPA@BMHOP-treated MCAO mice maintained their balance and walked in a straight line just as well as the Sham group, and showed significantly lower scores than other NPs-treated groups (Figure 4C).

NPs accumulated in cells with high levels of ROS, which triggered ROS-scavenging and antioxidative functions of RAPA@BMHOP. The released RAPA was anti-inflammatory and stimulated endothelium survival 70, resulting in a significant therapeutic effect in I/R injury. The therapeutic efficacy of NPs was subsequently assessed by the expression levels of pro-inflammatory factors (tumor necrosis factor-α (TNF-α) and interleukin 6 (IL-6) in the MCAO mouse model brain by ELISA. RAPA@BMHOP down-regulated pro-inflammatory factors and caused a significant increase in anti-inflammatory factors (IL-10) (Figure 4D-F). Also, mTOR inhibition with RAPA achieved similar anti-inflammatory effects, possibly by the direct activation of pro-inflammatory M1 phenotype microglia within the neurovascular unit to the anti-inflammatory M2 phenotype 71-73, attenuating neuroinflammation injury.

### Pharmacokinetic study *in vivo*

**Figure 5:**
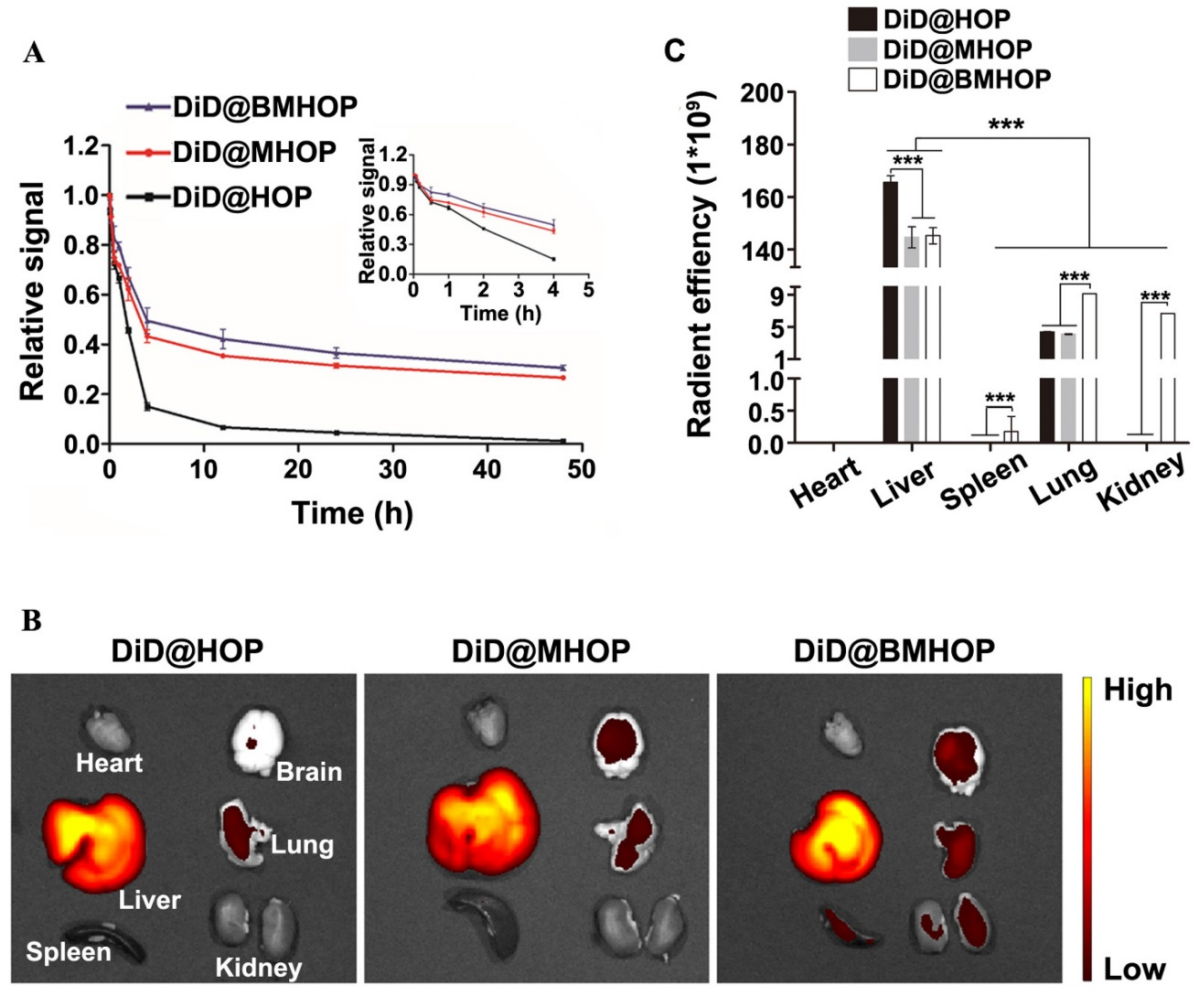
Pharmacokinetics and short-term *in vivo* evaluation of three different NPs. (A) *In vivo* pharmacokinetics of DiD@HOP, DiD@MHOP, and DiD@BMHOP (values represent mean ± SD, *n* = 3). (B) *Ex vivo* IVIS imaging of major organs from MCAO mice 12 h after i.v. injection of DiD@HOP, DiD@MHOP, and DiD@BMHOP. (C) Radiant efficiency of fluorescence intensity of different DiD@NPs in major organs after 12 h post-injection (values represent mean ± SD, *n* = 3, ***P < 0.001).

The *in vivo* circulation behavior of different NP formulations was evaluated in male C57 mice intravenously administered with 0.05 mg/kg NPs. Blood samples were taken at different time intervals, and the fluorescence intensity of the NPs in the plasma was assessed. As displayed in (Figure 5A), a l**o**nger circulation time (over 48 h) was observed in DiD@MHOP and DiD@BMHOP groups compared to the DiD@HOP group, which could be attributed to the immune evasion ability of the “do not eat me” protein CD47 on the bioengineered cell membranes. At 24 and 48 h, 36.5% and 30.6%, respectively, of the DiD@BMHOP remained in blood circulation; beyond this time, all NPs were rapidly eliminated, and none could be detected after 72 h. As expected, DiD@HOP showed much lower accumulation in the blood compared with the other groups, indicating the stealth capability of the cell membrane and confirming the successful coating of cell membranes onto the NPs, playing an important role in the predominant *in vivo* mimicking properties of the smart bioengineered NPs 21.

Physiologically-based pharmacokinetic evaluation has emerged as a credible strategy to predict *in vivo* drug distribution based on *ex vivo* analysis. To further analyze and validate that the bioengineered cell membrane-coated nanoplatforms improved the targeting ability and pharmacokinetics of nanomedicines, the *in vivo* stability and pharmacokinetics of three different NPs were evaluated in MCAO mice 12 h after the iv injection. Of the six organs analyzed, large amounts of DiD@NPs were accumulated in the liver, the main target organ mainly due to the reticuloendothelial system (Figure 5B). Also, macrophages are the primary cells responsible for the clearance of NPs due to their high prevalence in the liver. Cell membrane-coated NPs had promising robust pharmacokinetic and are well tolerated in mice. Thus, the prolonged systemic circulation might synergize with CXCR4 homing. Live imaging of *ex vivo* DiD@NPs in the major organs confirmed excellent cycling performance, and the pharmacokinetic analyses were consistent with DiD@BMHOP fluorescence (Figure 5C). The fluorescence in the lungs and kidneys was possibly due to elevated SDF-1 levels caused by the ischemia damage to these organs 36.

In summary, the CXCR4/SDF-1-targeting delivery strategy can be applied to improve the accumulation of administered drugs in the cerebral I/R injury area. RAPA@BMHOP had nearly a 3-fold higher targeting frequency than the other groups (Figure S9). This delivery system performed dual functions of the targeted delivery and "on-demand" cargo release 74,75.

### Safety assessment of nanocarriers *in vivo*

**Figure 6:**
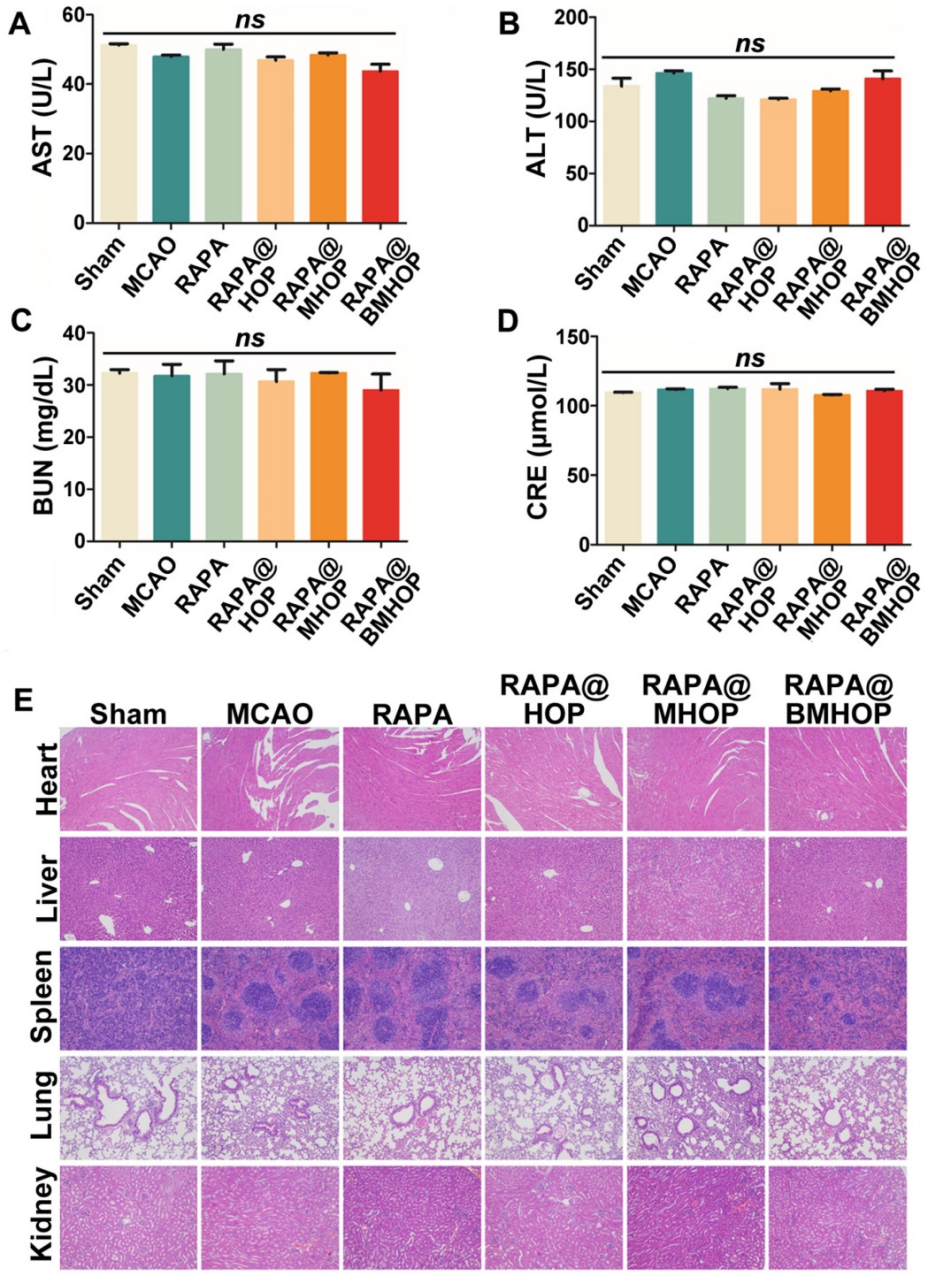
Assessing the biocompatibility of NPs in the treatment of cerebral I/R injury. (A-D) Biochemical parameters are indicative of hepatic and kidney functions: AST, ALT, BUN, and CRE. (values represent mean ± SD, *n* = 5, *ns*, no significance). (E) Histochemistry analysis of brain, heart, liver, spleen, lung, and kidney tissue sections stained with hematoxylin-eosin.

Biocompatibility must be carefully considered in applying nanocarriers *in vivo*. We evaluated the cytotoxicity of HOP, MHOP, and BMHOP at concentrations below 100 μg/mL in HUVECs by the MTS assay. The results showed no significant reduction in cell viability with different doses of three different NPs (Figure S12). To establish a preliminary *in vivo* safety profile, RAPA@HOP, RAPA@MHOP, and RAPA@BMHOP were injected into healthy mice via the tail vein, and serum biochemical analyses and histopathology evaluation were performed to monitor the potential toxicity. We found no significant abnormalities in various serum parameters (AST, aspartate aminotransferase; ALT, alanine aminotransferase; BUN, blood urea nitrogen; and creatinine levels) of renal and hepatic function after daily NP administration for 1 week (Figure 6A-D). H&E staining of liver, heart, spleen, kidney, and lung tissue sections following treatments with 5% sucrose, RAPA@HOP, RAPA@MHOP, and RAPA@BMHOP showed no evidence of abnormal and inflammatory cell infiltration (Figure 6E), demonstrating good biocompatibility of the nanoparticles *in vivo*.

## Conclusion

We developed RAPA@BMHOP, a smart bioengineered ROS-responsive nanoparticle formulation with targeted therapeutic and effective hydroxyl radical scavenging capability to treat I/R injury. This potential strategy was aimed at enhancing the active targeting of therapeutic agents, achieved by the interaction of the chemokine receptor CXCR4 with its ligand SDF-1 and prolonged *in vivo* circulation time. *In vitro* evaluation showed that direct reparation of I/R injury was achieved by combined ROS scavenging and anti-inflammatory effects, indicating RAPA@BMHOP's potential to provide protective effects. Furthermore, *ex vivo* fluorescence imaging showed that RAPA@BMHOP had remarkable active targeting functionalities and prolonged action time. Importantly, RAPA@BMHOP significantly ameliorated neuroscores and infarction volumes in response to surgical MCAO injury. Our findings suggested that, compared with current therapeutic tools, RAPA@BMHOP could provide better options for systemic control of multiple events during disease cascades and might be utilized as a potential strategy to enhance the clinical treatment of I/R injury.

## Methods

### Materials

*p-*Hydroxybenzyl alcohol (HBA), oxalyl chloride (OC), RAPA, and poly-(ethylene glycol)_2000_ (PEG_2000_) were obtained from Shanghai Aladdin Bio-Chem Technology Co, Ltd. (Shanghai, China). Tetrahydrofuran (THF), hydrogen peroxide (H_2_O_2_, 30%), DMSO, deuterium DMSO, and trichloromethane were purchased from Chongqing Chuandong chemical (group) Co, Ltd. (Chongqing, China). Polyclonal antibodies of CXCR4, CD47, and β-actin were acquired from Wuhan Sanying Biotechnology Co, Ltd. Proteintech Group. inc. (Wuhan, Hubei, China). Coomassie bright blue stain, BSA protein concentration tester, skim milk powder, TBST, cell protein extraction kit, DHE, DAPI, DiO, and DiD were purchased from Shanghai Beyotime biotechnology Co, Ltd. (Shanghai, China). SDF-1, ox-LDL, and AMD3100 were obtained from Beijing Solarbio Technology Co, Ltd. (Beijing, China). TTC and EB were acquired from Nanjing Jiancheng Bioengineering Institute. (Nanjing, Jiangsu, China). and Shanghai Yuanye Biotechnology Co, Ltd. (Shanghai, China), respectively.

### HOP synthesis

The copolyoxalate was synthesized by a one-step condensation; synthetic materials included polyethylene glycol-_2000_ (PEG_2000_), HBA, and OC. The polymerization was performed in dry THF under a low-temperature environment to generate the corresponding copolymers. The resulting copolyoxalate was purified through repeated precipitation in cold hexane, and after vacuum drying in an oven, a pale transparent solid was obtained. Using the encapsulation rate formula: EE = (The actual content of drug / The theoretical content of drug)*100%, the encapsulation efficiency (EE) of RAPA@HBA-OC-PEG_2000_ was 86%.

### LV5-CXCR4 (mouse) lentivirus construction and PMTAEC transfection

In preliminary experiments, the optimal transfection rate was determined as the highest multiplicity of infection (MOI=100). At different time points of 12, 24, 48, 72, and 96 h, the efficiency of transfection was measured by a fluorescence microscope. The expression levels of the target gene CXCR4 (mouse) was confirmed by using qPCR and Western blotting after establishing stable cell lines.

### Cell membrane-coated NPs

PMTAEC and RAPA@HOP cell membranes were fused to prepare RAPA@MHOP by an extrusion method. Briefly, cell membranes were extracted from PMTAEC, to which RAPA@HOP was added. Subsequently, the mixture solution was extruded using an Avestin mini-extruder (Avestin, LF-1, Canada) through a 200 nm polycarbonate porous membrane 10 times to harvest the RAPA@MHOP. The RAPA@BMHOP was prepared by the same protocol.

### *In vitro* drug release

The procedure used to study the drug release profile *in vitro* was as follows: RAPA@HOP, RAPA@MHOP, and RAPA@BMHOP nanoparticle solutions (1 mg/mL, 1 mL) in PBS (10 mL) were added to disposable dialysis cups (Slide-A-Lyzer MINI Dialysis Units, MWCO:3500 Da, Thermo Scientific). At different time points, the external drug release buffers were collected, and an equivalent amount of PBS was added.

### Detection of ROS *in vitro*

For the *in vitro* simulation experiment, activated HUVECs with H_2_O_2_-triggered upregulation of intracellular ROS were inoculated on the cell slide at 1×10^5^ cells per cm^2^ and cultured in 24-well plates. Subsequently, 5 μg/mL RAPA@BMHOP were added to each well, and the cells were cultured at 37 °C under 5% CO_2_. Next, 10 μM DHE as a superoxide anion fluorescence detection probe was added to each well at different time points of 0.5, 1, 2, 6 h during the treatment. Finally, after co-incubation with DHE for 20 min, the cells were resuspended in 1 mL of PBS and examined using flow cytometry (BD LSRFortessa).

### Animal modeling (the middle cerebral artery occlusion (MCAO) model)

Animal handling procedures were in accordance with the Ethics Committee of Chongqing Medical University for all *in vivo* experiments. Animal housing, care, and experiments were performed according to the guidelines and regulations of the Ethics Committee of Chongqing Medical University. We established the middle cerebral artery occlusion (MCAO) mouse model according to a previous protocol with minor modifications. The model mimicked I/R injury in ischemic stroke. Male C57 mice (24~28 g) were anesthetized with 4% pentobarbital sodium in saline via intraperitoneal injection. The external carotid artery (ECA) was exposed, and the monofilament was inserted into the internal carotid artery (ICA) through the ECA until it reached the middle cerebral artery (MCA), causing a blockage of blood flow. The MCAO monofilament was gently withdrawn 2 h later for reperfusion. Mice subjected to the same procedure without monofilament blocking were used as the sham group. Mice were housed separately in a temperature- and humidity-controlled room under a 12 h light-dark cycle with free access to food and water.

### TTC staining of the brain

Following tail vein injections for treatment, the cerebral infarct volume was measured by TTC staining, which shows the stained normal tissue in red and the unstained infarction in white. After the experiment, the mice were anesthetized with 5% chloral hydrate and decapitated. Brain tissue was quickly removed and rinsed with cold saline followed by refrigeration at -20 °C for 20 min. The tissues were sliced with a razor blade every 2 mm for TTC staining. The tissue sections were placed in a 12-well plate containing TTC staining solution and incubated at 37 °C in the dark for 15~20 min. The color changes of the samples were observed during incubation. Subsequently, the used TTC staining solution was removed, and the sections were fixed with 4% paraformaldehyde for 6 h for taking photographs.

### Statistical Analysis

A two-way analysis of variance (ANOVA) was performed to analyze data using GraphPad Prism 6.0. Values were expressed as means ± SD, and *P <* 0.05 was considered statistically significant.

## Supplementary Material

Supplementary methods and figures.

## Abbreviations

I/R: ischemia-reperfusion
CXCR4: C-X-C motif chemokine receptor 4
SDF-1: stromal cell derived factor-1
HOP: HBA-OC-PGE_2000_
RAPA: rapamycin
RAPA@HOP: HOP loading with RAPA
RAPA@MHOP: normal cell membrane coated HOP, loading with RAPA
RAPA@BMHOP: bioengineered CXCR4-overexpressed cell membrane coated nanoparticles HOP, loading with RAPA
MCAO: the middle cerebral artery occlusion model mouse
PI: Propidium Iodide
DiD: 1,1-dioctadecyl-3,3,3,3-tetramethylindodicarbocyanine
DMSO: dimethyl sulfoxide
DiD@BMHOP: bioengineered CXCR4-overexpressed cell membrane coated nanoparticles HBA-OC-PEG_2000_, loading with DiD
OC: oxalyl chloride
DiO: 3,3'-dioctadecyloxacarbocyanine perchlorate
DLS: dynamic light scattering
DMSO: dimethyl sulfoxide
ELISA: enzyme linked immunosorbent assay
FCM: flow cytometry
GFP: green fluorescent protein
PBS: phosphate buffer solution
MTS: MTS cell proliferation colorimetric assay kit
q-PCR: real-time quantitative PCR detecting system
TEM: transmission electron microscopy
TTC: 2,3,5-triphenyl tetrazolium chloride
ECA: external carotid artery
ICA: internal carotid artery
MCA: middle cerebral artery
DHE: dihydroethidium
CLSM: confocal laser scanning microscope
DAPI: 4,6-diamidino-2-phenylindole
ROS: reactive oxygen species
PEG: polyethylene glycol
PMTAEC: primary mouse thoracic aorta endothelial cells
CM: normal cell membrane
HCM: CXCR4-overexpressed cell membrane

## References

[1] Hankey GJ (2017). Stroke. The Lancet.

[2] Nomura E, Kohriyama T, Kozuka K, Kajikawa H, Nakamura S, Matsumoto M (2004). Significance of serum soluble thrombomodulin level in acute cerebral infarction. Eur J Neurol.

[3] Deguchi I, Dembo T, Fukuoka T, Nagoya H, Maruyama H, Kato Y (2012). Usefulness of MRA-DWI mismatch in neuroendovascular therapy for acute cerebral infarction. Eur J Neurol.

[4] Teng YM, Jin HQ, Nan D, Li MN, Fan CH, Liu YY (2018). In vivo evaluation of urokinase-loaded hollow nanogels for sonothrombolysis on suture embolization-induced acute ischemic stroke rat model. Bioact Mater.

[5] Wang J, Li XL, Song Y, Su QF, Xiakeerzhati X, Yang W (2021). Injectable silk sericin scaffolds with programmable shape-memory property and neuro-differentiation-promoting activity for individualized brain repair of severe ischemic stroke. Bioact Mater.

[6] Chan Pak H (1996). Role of Oxidants in Ischemic Brain Damage. Stroke.

[7] Allen CL, Bayraktutan U (2009). Oxidative stress and its role in the pathogenesis of ischaemic stroke. Int J Stroke.

[8] D'Autréaux B, Toledano MB (2007). ROS as signaling molecules: mechanisms that generate specificity in ROS homeostasis. Nat Rev Mol Cell Biol.

[9] Salim S (2017). Oxidative stress and the central nervous system. J Pharmacol Exp Ther.

[10] Yoshida H, Yanai H, Namiki Y, Fukatsu-Sasaki K, Furutani N, Tada N (2006). Neuroprotective effects of edaravone: a novel free radical scavenger in cerebrovascular injury. CNS Drug Rev.

[11] Christoph J B, Nikolina PM, Joseph L W (2016). Innate sensing of oxidation-specific epitopes in health and disease. Nat Rev Immunol.

[12] Clemens DL, Dahn D, Michael S, Cleofes C, Andrew M, Jacob H (2018). Novel antioxidant properties of doxycycline. Int J Mol Sci.

[13] Shcharbina N, Shcharbin D, Bryszewska M (2013). Nanomaterials in stroke treatment: perspectives. Stroke.

[14] Niu XQ, Chen JJ, Gao JQ (2018). Nanocarriers as a powerful vehicle to overcome blood-brain barrier in treating neurodegenerative diseases: focus on recent advances. Asian J Pharm Sci.

[15] Fontana F, Shahbazi MA, Liu D, Hongbo Z, Ermei M, Jarno S (2017). Multistaged nanovaccines based on porous silicon@acetalated dextran@cancer cell membrane for cancer immunotherapy. Adv Mater.

[16] Wang Y, Zhang K, Li TH, Ali M, Qin X, Luo L (2021). Macrophage membrane functionalized biomimetic nanoparticles for targeted anti-atherosclerosis applications. Theranostics.

[17] Fang RH, Kroll AV, Gao W, Zhang LF (2018). Cell Membrane Coating Nanotechnology. Adv Mater.

[18] CheMing J H, Ronnie H F, KueiChun W, Brian T L, Soracha T, Diana D (2015). Nanoparticle biointerfacing by platelet membrane cloaking. Nature.

[19] Reddy MK, Labhasetwar V (2009). Nanoparticle-mediated delivery of superoxide dismutase to the brain: an effective strategy to reduce ischemia-reperfusion injury. FASEB J.

[20] Zhang XB, Xiong JC, Wang KY, Yu H, Sun BJ, Ye H (2021). Erythrocyte membrane-camouflaged carrier-free nanoassembly of FRET photosensitizer pairs with high therapeutic efficiency and high security for programmed cancer synergistic phototherapy. Bioact Mater.

[21] CheMing J H, Li Z, Santosh A, Connie C, Ronnie H F, Zhang LF (2011). Erythrocyte membrane-camouflaged polymeric nanoparticles as a biomimetic delivery platform. Proc Natl Acad Sci U S A.

[22] Wang Y, Zhang K, Qin X, Li TH, Qiu JH, Yin TY (2019). Biomimetic nanotherapies: red blood cell based core-shell structured nanocomplexes for atherosclerosis management. Adv Sci (Weinh).

[23] Jennie B, Deirdre M, Bernadine M F, Sushil D, Stefan G, Gunnar H (2020). Wood hemicelluloses exert distinct biomechanical contributions to cellulose fibrillar networks. Nat Commun.

[24] Simon F D M, Karen V, Marinee K C, Inge P, Veerle G, Robert P H (2006). Phenotypic correction of von Willebrand disease type 3 blood-derived endothelial cells with lentiviral vectors expressing von Willebrand factor. Blood.

[25] Bowers M, Zhang B, Ho Y, Agarwal P, Chen C C, Bhatia R (2015). Osteoblast ablation reduces normal long-term hematopoietic stem cell self-renewal but accelerates leukemia development. Blood.

[26] Christoph M, Tina C, Susanne R, Astrid M, Franziska H, Lucas H (2020). Cryopreservation impairs 3-D migration and cytotoxicity of natural killer cells. Nat Commun.

[27] Mascarenhas MI, Parker A, Dzierzak E, Ottersbach K (2009). Identification of novel regulators of hematopoietic stem cell development through refinement of stem cell localization and expression profiling. Blood.

[28] Kumar DL, DeFalco T (2018). A perivascular niche for multipotent progenitors in the fetal testis. Nat Commun.

[29] Yvernogeau L, Klaus A, Maas J, Ismaël MP, Bart W, Stefan SM (2020). Multispecies RNA tomography reveals regulators of hematopoietic stem cell birth in the embryonic aorta. Blood.

[30] Kim H (2012). Will the Stroma-derived Factor-1α (CXCL12)/CXCR4 Pathway Become a Major Concern for Advanced Colorectal Cancer?. J Korean Soc Coloproctol.

[31] Meizhang L, Richard MR (2009). The roles of chemokine CXCL12 in embryonic and brain tumor angiogenesis. Semin Cancer Biol.

[32] Karin N (2010). The multiple faces of CXCL12 (SDF-1alpha) in the regulation of immunity during health and disease. J Leukoc Biol.

[33] Cencioni C, Melchionna R, Straino S, Marta R, Claudia C, Valentina A (2013). Ex vivo acidic preconditioning enhances bone marrow ckit+ cell therapeutic potential via increased CXCR4 expression. Eur Heart J.

[34] Hoggatt J, Singh P, Sampath J, Pelus LM (2009). Prostaglandin E2 enhances hematopoietic stem cell homing, survival, and proliferation. Blood.

[35] Basu S, Broxmeyer HE (2005). Transforming growth factor-β1 modulates responses of CD34+ cord blood cells to stromal cell-derived factor-1/CXCL12. Blood.

[36] Liu B, Li ZH (2008). Endoplasmic reticulum HSP90b1 (gp96, grp94) optimizes B-cell function via chaperoning integrin and TLR but not immunoglobulin. Blood.

[37] Jessy D, Sifeng C, Sergio C, Anna GP, Halina W, Sergio LC (2007). Stromal cell-derived factor 1 promotes angiogenesis via a heme oxygenase 1-dependent mechanism. J Exp Med.

[38] Ma JN, Zhang SQ, Liu J, Liu FY, Du FY, Li M (2019). Targeted drug delivery to stroke via chemotactic recruitment of nanoparticles coated with membrane of engineered neural stem cells. Small.

[39] Dabrowska S, Andrzejewska A, Lukomska B, Janowski M (2019). Neuroinflammation as a target for treatment of stroke using mesenchymal stem cells and extracellular vesicles. J Neuroinflammation.

[40] Oldenborg PA, Zheleznyak A, Fang YF, Lagenaur CF, Gresham HD, Lindberg FP (2000). Role of CD47 as a marker of self on red blood cells. Science.

[41] Schweitzer C, Schmidt R (2003). Physical mechanisms of generation and deactivation of singlet oxygen. Chem Rev.

[42] Nosaka Y, Nosaka AY (2017). Generation and detection of reactive oxygen species in photocatalysis. Chem Rev.

[43] Lianbing Z, Linda L, Wolfram M, Eckhard P, Ulrich G (2010). Reducing stress on cells with apoferritin-encapsulated platinum nanoparticles. Nano Lett.

[44] Zhang W, Hu SL, Yin JJ, He WW, Lu W, Ma M (2016). Prussian blue nanoparticles as multienzyme mimetics and reactive oxygen species scavengers. J Am Chem Soc.

[45] Mu JS, Zhao X, Li J, Yang EC, Zhao XJ (2016). Novel hierarchical NiO nanoflowers exhibiting intrinsic superoxide dismutase-like activity. J Mater Chem B.

[46] Hyunjin P, Soojin K, Sujin K, Yiseul S, Kyungryul S, Donghyun H (2010). Antioxidant and anti-inflammatory activities of hydroxybenzyl alcohol releasing biodegradable polyoxalate nanoparticles. Biomacromolecules.

[47] Tao WH, He ZG (2018). ROS-responsive drug delivery systems for biomedical applications. Asian J Pharm Sci.

[48] Liu J, Mori A (1993). Antioxidant and pro-oxidant activities of p-hydroxybenzyl alcohol and vanillin: effects on free radicals, brain peroxidation and degradation of benzoate, deoxyribose, amino acids and DNA. Neuropharmacology.

[49] Crino PB (2016). The mTOR signaling cascade: paving new roads to cure neurological disease. Nat Rev Neurol.

[50] Tanaka M, Sackmann E (2005). Polymer-supported membranes as models of the cell surface. Nature.

[51] Cheming J H, Ronnie H F, Jonathan C, Brian L, Liangfang Z (2013). A biomimetic nanosponge that absorbs pore-forming toxins. Nat Nanotechnol.

[52] Du XR, Huang YK, Pan XL, Han B, Su Y, Jiang QK (2020). Size-dependent strong metal-support interaction in TiO(2) supported Au nanocatalysts. Nat Commun.

[53] Xu JC, Zhang YL, Xu JQ, Liu GN, Di CZ, Zhao X (2020). Engineered nanoplatelets for targeted delivery of plasminogen activators to reverse thrombus in multiple mouse thrombosis models. Adv Mater.

[54] Jiang Q, Luo ZM, Men YZ, Yang P, Peng HB, Guo RR (2017). Red blood cell membrane-camouflaged melanin nanoparticles for enhanced photothermal therapy. Biomaterials.

[55] Rebres RA, Kajihara K, Brown EJ (2005). Novel CD47-dependent intercellular adhesion modulates cell migration. J Cell Physiol.

[56] Chen WS, Zeng K, Liu H, Ouyang J, Wang LQ, Liu Y (2017). Cell membrane camouflaged hollow prussian blue nanoparticles for synergistic photothermal-/chemotherapy of cancer. Adv Funct Mater.

[57] Rao L, Cai B, Bu LL, Liao QQ, Guo SS, Zhao XZ (2017). Microfluidic electroporation-facilitated synthesis of erythrocyte membrane-coated magnetic nanoparticles for enhanced imaging-guided cancer therapy. ACS Nano.

[58] Lauri B, Marco M, Marie L, Gary B, Ron Ma, Simon F (2005). Durable engraftment of AMD3100-mobilized autologous and allogeneic peripheral-blood mononuclear cells in a canine transplantation model. Blood.

[59] Elena D F, Daniele P, Anna R T, Stefania S, Maria G I, Alessia O (2004). SDF-1 involvement in endothelial phenotype and ischemia-induced recruitment of bone marrow progenitor cells. Blood.

[60] Mohamed H, Achim K, El M Q (2012). Shedding new light on neurodegenerative diseases through the mammalian target of rapamycin. Prog Neurobiol.

[61] Li MC, Yu J, Li Y, Li DJ, Yan D, Qu ZL (2010). CXCR4 positive bone mesenchymal stem cells migrate to human endothelial cell stimulated by ox-LDL via SDF-1α/CXCR4 signaling axis. Exp Mol Pathol.

[62] Jacob S B, Daniel C P, Jacob W M, Oscar A M, Carlos H V, Priyal P (2018). Red blood cell-hitchhiking boosts delivery of nanocarriers to chosen organs by orders of magnitude. Nat Commun.

[63] Johanna S, Kristin N B, Jens L, Till O, Volker M, Katharina L (2019). Noncovalent targeting of nanocarriers to immune cells with polyphosphoester-based surfactants in human blood plasma. Adv Sci (Weinh).

[64] Kin M A, Steven I P, Andrew Z W (2020). Trispecific natural killer cell nanoengagers for targeted chemoimmunotherapy. Sci Adv.

[65] Sandoval KE, Witt KA (2008). Blood-brain barrier tight junction permeability and ischemic stroke. Neurobiol Dis.

[66] Zahraa S A, Dhifaf J, Sabahuddin S A, Raymond W, Michael H, Graham C (2019). Selective liposomal transport through blood brain barrier disruption in ischemic stroke reveals two distinct therapeutic opportunities. ACS Nano.

[67] Changsun K, Sian G, Chulgyu S, Peter M K, Seong P K, Jonghu J (2017). Fibrin-targeted and H_2_O_2_-responsive nanoparticles as a theranostics for thrombosed vessels. ACS Nano.

[68] Laura N, Ruja T, Arianna V, Cinzia M, Francesca R, Tiziana B (2007). Reactive oxygen species plasmatic levels in ischemic stroke. Mol Cell Biochem.

[69] Gong WH, Zheng WX, Wang J, Chen SH, Pang B, Xiamin H (2012). Coexistence of hyperlipidemia and acute cerebral ischemia/reperfusion induces severe liver damage in a rat model. World J Gastroenterol.

[70] Wang P, He YT, Li DJ, Han RR, Liu GY, Kong DX (2016). Class I PI3K inhibitor ZSTK474 mediates a shift in microglial/macrophage phenotype and inhibits inflammatory response in mice with cerebral ischemia/reperfusion injury. J Neuroinflammation.

[71] Michalis P, Gina H, Maria X, Lisa C H, Simon N, M Mary M (2013). Tsc1 (hamartin) confers neuroprotection against ischemia by inducing autophagy. Nat Med.

[72] Shiqi W, Saowanee W, Patrícia F, Giuseppina M, Yaping D, Alexandra C (2020). Intracellular delivery of budesonide and polydopamine co-loaded in endosomolytic poly(butyl methacrylate-co-methacrylic acid) grafted acetalated dextran for macrophage phenotype switch from M1 to M2. Adv Ther (Weinh).

[73] Lin ZJ, Shen DN, Zhou WX, Zheng YF, Kong TT, Liu XY (2021). Regulation of extracellular bioactive cations in bone tissue microenvironment induces favorable osteoimmune conditions to accelerate in situ bone regeneration. Bioact Mater.

[74] Guo X, Deng G, Liu J, Zou P, Du FY, Liu FY (2018). Thrombin-responsive, brain-targeting nanoparticles for improved stroke therapy. ACS Nano.

[75] Tsou YH, Zhang XQ, Zhu H, Sahla S, Xu XY (2018). Drug delivery to the brain across the blood-brain barrier using nanomaterials. Small.

