# Supplementary material for: Bioengineering CXCR4-overexpressing cell membrane functionalized ROS-responsive nanotherapeutics for targeting cerebral ischemia-reperfusion injury
Source: Theranostics. 2021 Jul 6;11(16):8043–56. doi: 10.7150/thno.60785 (PMC8315061; doi:10.7150/thno.60785)
Supplement: Supplementary file 1 — Supplementary methods and figures. [file thnov11p8043s1.pdf]

Supporting Information for

Bioengineering CXCR4-overexpressing cell membrane functionalized ROS-responsive nanotherapeutics for targeting cerebral ischemia-reperfusion injury

Li Luo[1], Guangchao Zang[2], Boyan Liu[1], Xian Qin[1], Yuchan Zhang[3], Yidan Chen[1], Haijun Zhang[4], Wei Wu[1]✉, and Guixue Wang[1]✉

1 Key Laboratory for Biorheological Science and Technology of Ministry of Education, State and Local Joint Engineering Laboratory for Vascular Implants, Bioengineering College of Chongqing University, Chongqing University, Chongqing 400044, China.

2 Laboratory of Tissue and Cell Biology, Lab Teaching & Management Center, Chongqing Medical University, Chongqing, 400016, China.

3 Institute of Life Science, Chongqing Medical University, Chongqing, 400016, China.

4 Department of Vascular & Intervention, Tenth Peoples' Hospital of Tongji University, Shanghai, 200072, China

Cell lines and animals

Primary mouse thoracic aorta endothelial cells (PMTAEC) were isolated and cultured from C57BL/6 mice, mouse mononuclear macrophage leukemia cell (RAW264.7) and Human umbilical vein endothelial cells were obtained from Type Culture Collection of Chinese Academy of Sciences (Shanghai, China). PMTAEC cells were cultured in primary endothelial cell medium, RAW264.7 cells were cultured in DMEM medium and HUVEC cells were cultured in RPMI 1640 medium at 37 °C in a 5% CO_2 humidified incubator. All media contained 10% FBS, 100 U mL^{-1} penicillin and 100 μg mL^{-1} streptomycin. Eight-week old C57BL/6 mice (male) were purchased from SPF (Beijing) Biotechnology Co, Ltd. (Beijing, China).

Cell culture

PMTAEC cells were cultured by primary cell complete culture medium (ScienceCell. Beijing Yuhengfeng Biotech Co, Ltd.), including 500 mL of basal medium, 25 mL of FBS (fetal bovine serum), 5 mL of endothelial cell growth supplement and 5 mL of penicillin/streptomycin solution, in the 37 ^{o}C 5% CO_2 saturated humidity incubator.

Lentivirus construction

The lentivirus vector (A5585-1) for CXCR4 (mouse) overexpression were designed and chemically synthesized from Shanghai GenePharma Co, Ltd. (Shanghai China). The sequence of target gene CXCR4 comes from Genebank accession NO: NM_009911.3. The lentiviral vector with green fluorescence protein (GFP) was used as the identification of successful transfection marker.

The extraction of cell membrane

Reagents A melt at room temperature and PMSF (Phenylmethylsulfonyl fluoride) was added. A few minutes before use to make the final concentration of PMSF 1 mM. Cultured about 20-50 million cells, washed them with PBS, and scraped the cells with a cell scraper. Cells were collected by centrifugation, and cell precipitation was left for later use after supernatant was removed. Added 1 mL working solution to 20-50 million cells, gently and fully suspend the cells, and left in an ice bath for 10-15 min. The samples were successively frozen and thawed three times in liquid nitrogen and room temperature. Removed nuclei and unfragmented cells: centrifuged at 4 ºC, 700g, for 10 min. Carefully collected the supernatant into a new centrifuge tube. When the supernatant was absorbed, did not contact with the precipitation to precipitate cell membrane fragments: centrifuged at 4 ºC for 14000×*g* for 30 min to precipitate cell membrane fragments.

Preparation of NPs

HBA-OC-PEG_{2000} (HOP) was prepared by chemical synthesis. Briefly, HBA, PEG_{2000} were dissolved into CH_2Cl_2 (methylene chloride). Mixed the dissolved HBA with PEG_{2000}, and the whole reaction process must be operated at low temperature (in an ice bath), OC (oxaloyl chloride) was rapidly added into the above mixture solution. It had been reacted at room temperature and stayed overnight, then the HOP was obtained. RAPA@HOP was prepared by nanoprecipitation method. HOP and RAPA were respectively dissolved in the tetrahydrofuran (THF), under the action of full agitation, appropriate amount of RAPA THF solution was dropped into the HOP THF

solution which were mixed with H_2O. The mixture was further dialyzed using dialysis bag (molecular weight cut-off, MWCO: 3500 Da) against water to remove the free RAPA and THF.

The construction of intracellular ROS overexpression

The cells were plated at a density of 5×10^5 cells/well in six-well Biocoat plates and grown for 24 h in complete medium that 1640 culture medium supplemented with 10% fetal bovine serum (FBS), at 37 °C in a normoxia with 5% CO_2 atmosphere. Cells were washed twice in 1640 without FBS (Life Technologies, Carlsbad, CA, USA) switched to 1640 without FBS supplemented with 300 μM H_2O_2 and placed in modular incubator chambers (BillupsRothenberg, Del Mar, CA, USA) for 24 h.

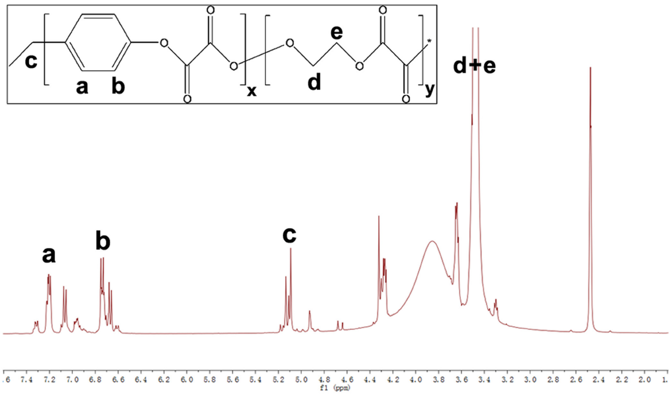

Figure S1. The NMR results of HOP. The chemical structure of polymers was identified with a 400 MHz ^{1}H NMR spectrometer (Bruker ADVANCE500). ^{1}H NMR in deuterated DMSO-d_6 on a 400 MHz spectrometer. The chemical structure of HOP was confirmed by the ^{1}H NMR. Comparing it to the resonance signal of the integrated methoxy group of the PEG_{2000} chain at ~3.5 ppm, the resonance peaks at ~4.3 ppm correspond to the methylene protons adjacent to oxalate ester linkages, two multiplet aromatic proton peaks appear at ~6.7 and ~7.2 ppm. These results demonstrated successful polymerization from the condensation reaction between OC and six PEG_{2000} and one HBA, generating polyoxalate containing peroxalate ester linkages.

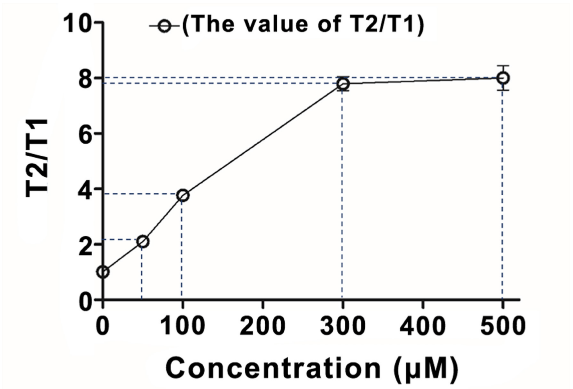

Figure S2. The ratio of changes in nanoparticles size at beginning to 12 h later with increasing H_2O_2 concentrations [every size was analyzed three times for each sample,

and there was no significant difference between the results (intra-group analysis), but between-group comparisons demonstrated that significant differences exists between the groups ≥ 300 μM and groups < 300 μM; T1: the nanoparticle size after 10 min; T2: the nanoparticle size after 12 h].

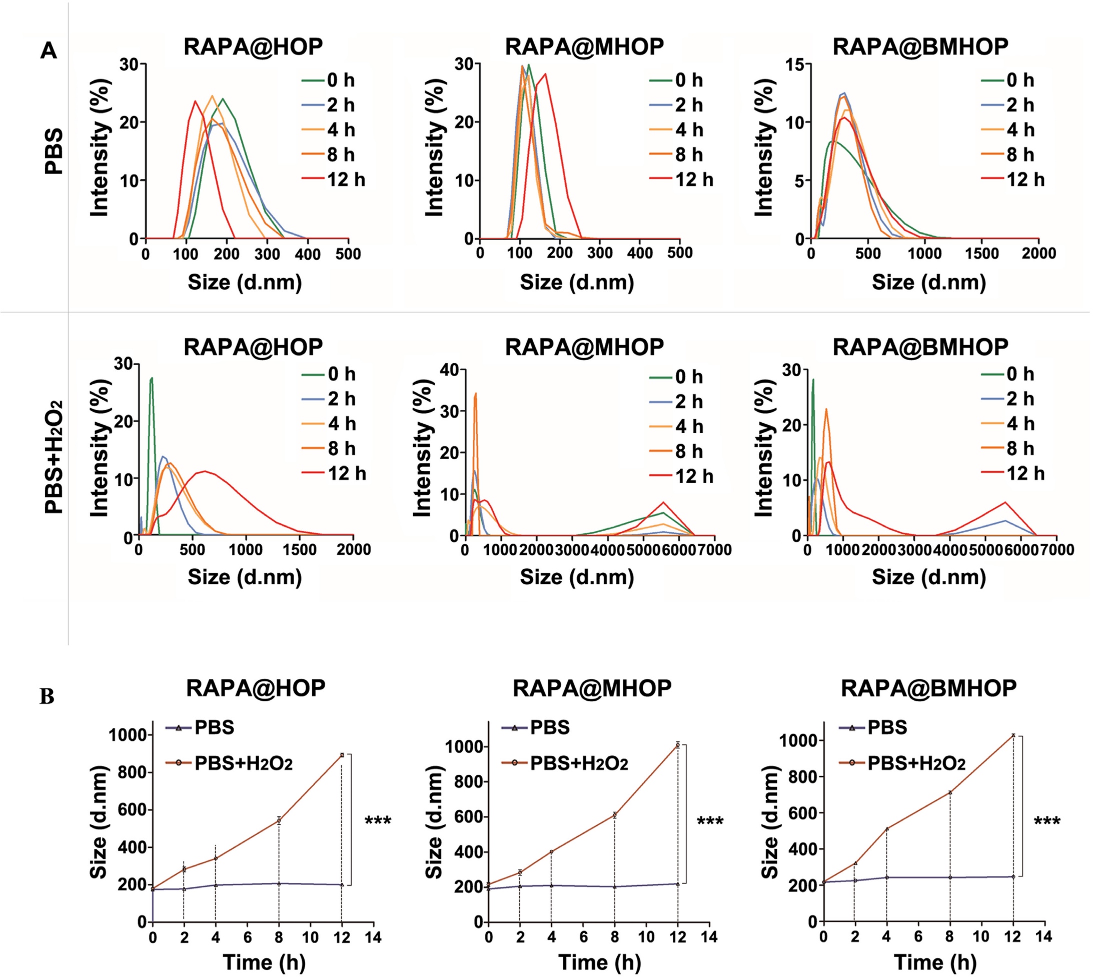

Figure S3. (A) The DLS results of nanoparticles in PBS and in PBS containing H_2O_2. (B) Quantitative comparison of nanoparticle diameters changes over time in different treatments (in PBS and in PBS containing H_2O_2, ****P* < 0.001).

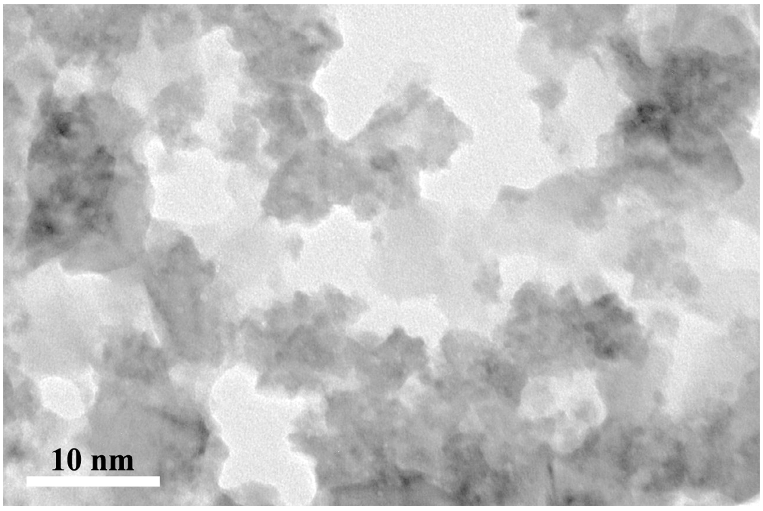

Figure S4. The TEM image of RAPA@BMHOP nano fragments after degradation (scale bar = 10 nm).

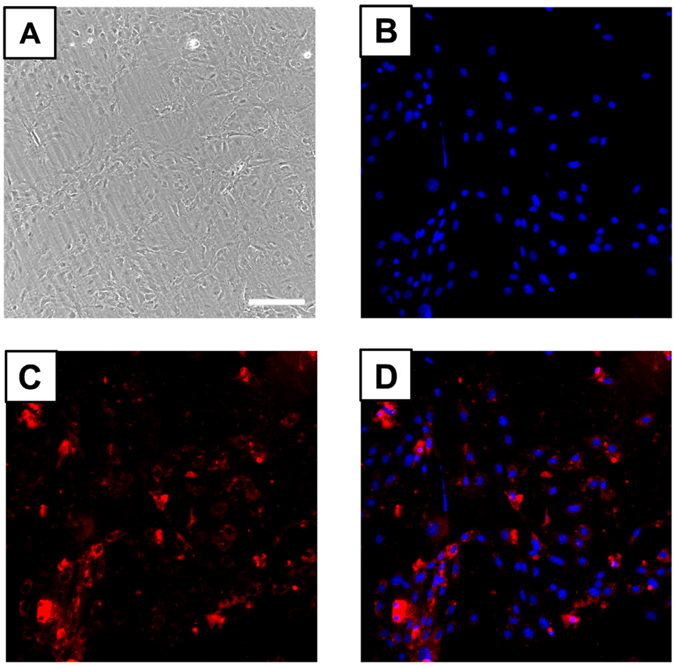

Figure S5. Primary mouse thoracic aortic endothelial cells were extracted and the cell properties were identified by immunofluorescence: CD31 (the endothelial cell specific marker protein). (A) The third generation cell original figure; (B) DAPI for nuclear staining; (C) immunofluorescence for CD31; (D) merge for endothelial cell identification (scale bar = 20 μm).

Virus infection:

PBS

Trypsin-EDTA Solution (ScienceCell)

24 plate well (Corning)

Lentivirus-NC virus solution (GenePharma, 1×10^9 TU/mL)

Step:

1). The well-grown MTAEC cells were digested and re-suspended, and an appropriate amount of cells were inoculated into a 24-well plate and placed in a 37 ^{o}C incubator overnight

2). The negative control virus was diluted with the medium at 1:10, 1:100, 1:1000, and the total volume was about 500 μL. Polybrene was added with a final concentration of 5 μg/mL

3). The original culture medium was removed from the 24-well plate and replaced with negative control virus gradient diluting solution

4). After 24 h, the diluent of negative control virus was removed and replaced with 500 μL fresh medium

5). The results were observed and recorded 48 hours later under an inverted fluorescence microscope

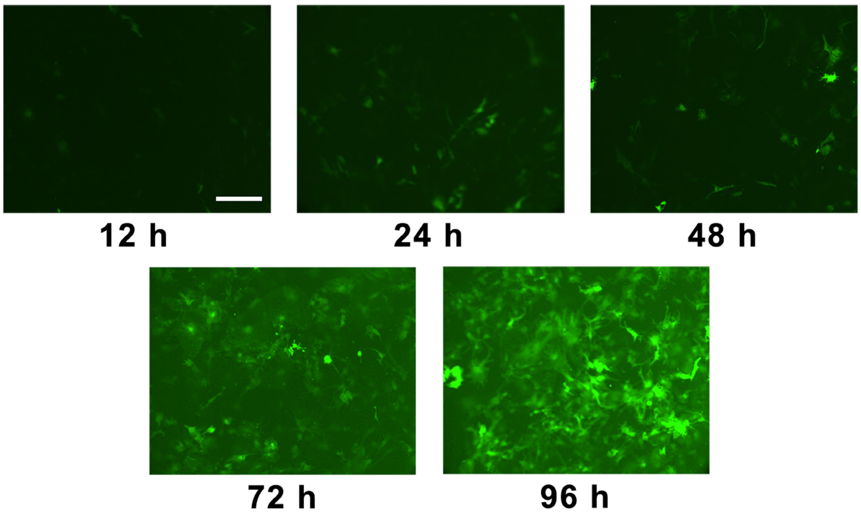

Figure S6. Identification of lentivirus transfection rate in cells. Following the experimental instructions, CXCR4 was successfully overexpressed in cells after transfection for 96 h at MOI=100. The transfection effect from 5 different time periods was shown in the above figure:12 h, 24 h, 48 h, 72 h, 96 h , and GFP was the green fluorescence indicator (scale bar = 20 μm).

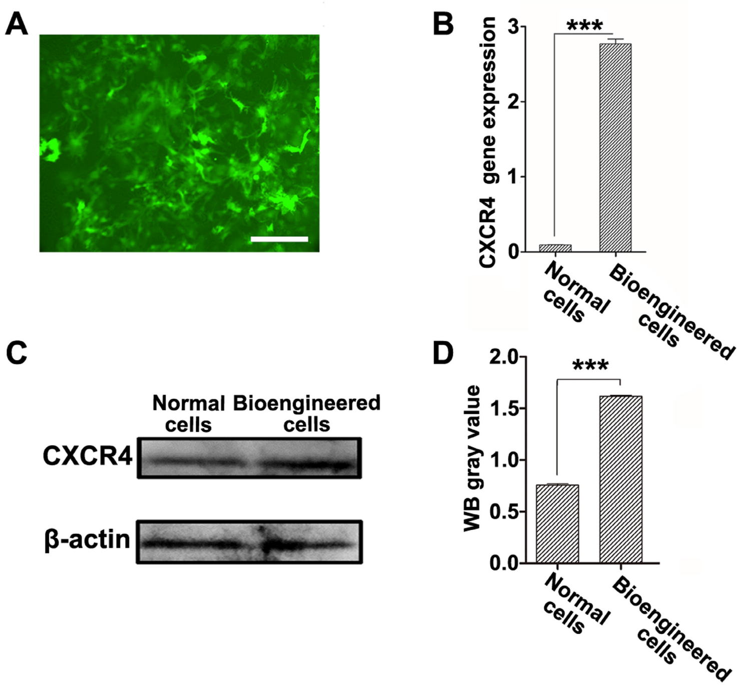

Figure S7. Cells that are called bioengineered cells after lentiviral transcription, theoretically, the transfected cells can obtain stable target protein expression for a long time in progeny cells of passage and culture. The DNA of the bioengineered primary cells were extracted and comparing with the unmodified cells for detection by q-PCR, and it was found that CXCR4 had significant high expression at the molecular level. Meanwhile, the expression level of CXCR4 was confirmed by using western blotting. (A) The results of q-PCR (values represent mean ± SD, n = 3, ***P < 0.001). (B) GPF of the bioengineered primary cells observed by microscopy. (C) The WB results of CXCR4 expression. (D) The analysis of WB gray value (scale bar = 20 μm; values represent mean ± SD, n = 3, ***P < 0.001).

Cell migration experiment：

1). Mouse thoracic aortic endothelial cells and bioengineering cells were respectively removed from the culture flashs and resuspended at 0.5×10^6 cells/mL in serum free culture medium.

2). SDF-1(100 ng/mL) was placed in the lower wells. Test cells were then placed in the upper chamber for 60 min.

3). After incubation, the upper surface of the transwell membrane was wiped gently with a cotton swab to remove non-migrating cells. Cells which migrated to the lower surface of the membrane were stained using DAPI.

4). Migrated cells were counted in 3 different fields of a defined size (5×0.25 mm^2) using a phase contrast microscope and the mean cellular migration rate was calculated.

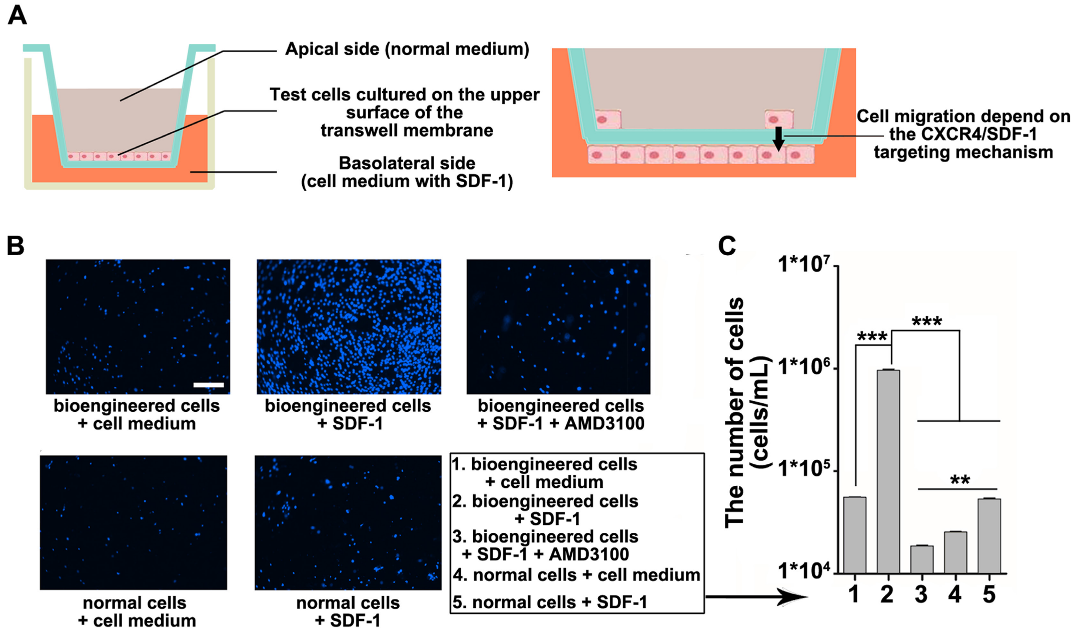

Figure S8. In order to verify the target ability of bioengineered cells, the 6-well cell culture plates transwell chamber (aperture 8 μm) were performed on cell migration experiments to evaluate it. This cell migration experiment mechanism: transwell chambers located in the culture plate, due to the permeability of polycarbonate membrane between apical side and basolateral side, the ingredients of the lower culture medium can affect the cell movement in the upper chamber. Appropriate SDF-1 (100 ng/mL) was added to the lower culture medium as a chemokine for the cells on apical side, it was observed that some cells migrated to the basolateral side of the membrane after 1 h. In contrast, (A) The scheme of transwell cell migration experiments. (B) four other groups for experimental control were set: bioengineered cells + culture medium (without SDF-1); bioengineered cells + SDF-1 (100 ng/mL) ; bioengineered cells + SDF-1(100 ng/mL) + AMD 3100(100 ng/mL): Plerixafor (AMD 3100) is a selective CXCR4 antagonist; normal cells + culture medium (without SDF-1); normal cells + SDF-1(100 ng/mL); (C) The analysis of cell

fluorescence concentration. However, comparing with treatment group there is no significant cell migration occurred (scale bar = 20 μm; values represent mean ± SD, n = 3,***P < 0.001).

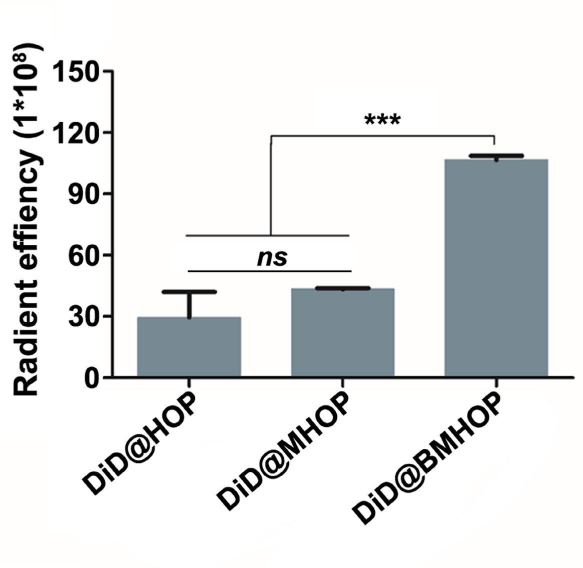

Figure S9. Quantification of the radiant efficiency of NPs in the brain by IVIS Lumina imaging software performed by drawing a region of interest (ROI) (values represent mean ± SD, n = 3, ***P < 0.001, *ns*, no significance).

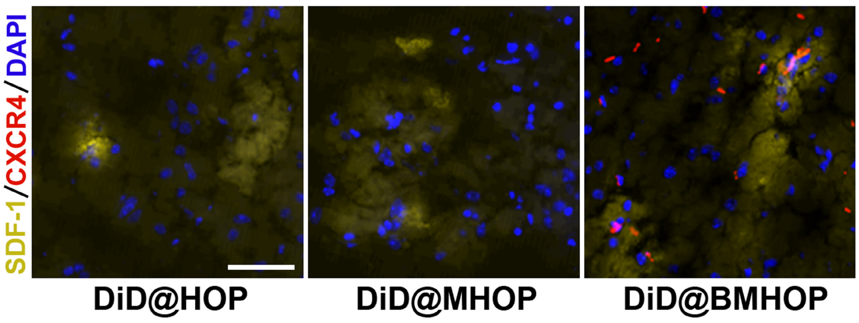

Figure S10. Images of immunofluorescence staining analysis of brain section for three different DiD@NPs (white scale bar = 100 μm; blue: DAPI for nucleus; yellow: SDF-1 overexpression within brain damaged tissue; red: DiD@NPs).

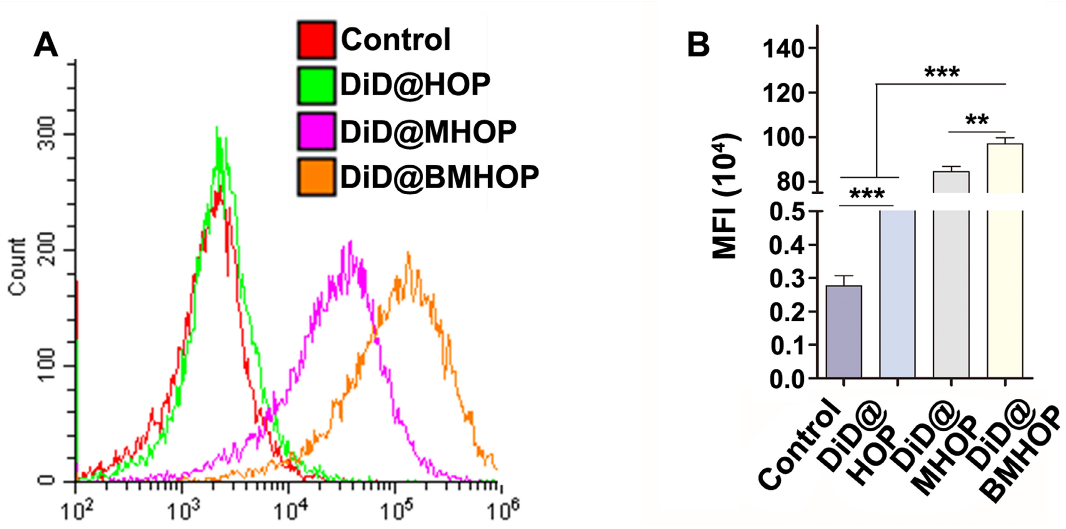

Figure S11. (A) The intracellular ROS production after DiD@BMHOP treatement at different time points weas measured by flow cytometry. (B) The quantitative analysis of red fluorescence of DiD@BMHOP at different time points (values represent mean ± SD, $n = 3$, $^{**}P < 0.01$ and $^{***}P < 0.001$, *ns*, no significance).

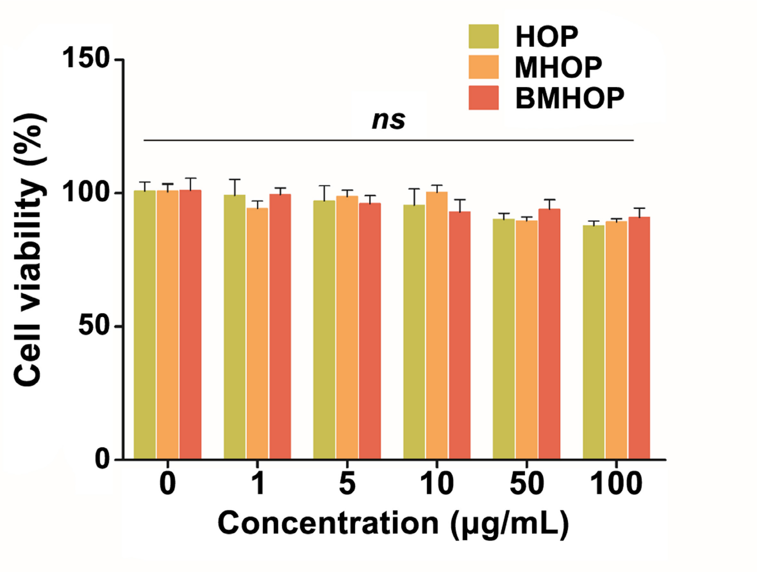

Figure S12. The cytotoxicity of HOP, MHOP and BMHOP acting on endothelial cells were detected by MTS method (values represent mean ± SD, $n = 6$, *ns*, no significance).
